# Supplementary material for: Hydrovoltaic effect-enhanced photocatalysis by polyacrylic acid/cobaltous oxide–nitrogen doped carbon system for efficient photocatalytic water splitting
Source: Nat Commun. 2023 Mar 30;14:1759. doi: 10.1038/s41467-023-37366-3 (PMC10063643; doi:10.1038/s41467-023-37366-3)
Supplement: Supplementary file 1 — Supplementary Information [file 41467_2023_37366_MOESM1_ESM.pdf]

Supplementary Information for

Hydrovoltaic effect-enhanced photocatalysis by polyacrylic acid/cobaltous oxide-nitrogen doped carbon system for efficient photocatalytic water splitting

Xu Xin[1], Youzi Zhang[1], Ruiling Wang[1], Yijin Wang[1], Peng Guo[1], Xuanhua Li[1*]

[1] State Key Laboratory of Solidification Processing, Center for Nano Energy Materials, School of Materials Science and Engineering, Northwestern Polytechnical University, Xi'an 710072, China

[*]Corresponding author E-mail: lixh32@nwpu.edu.cn

This PDF file includes:
Supplementary Texts
Supplementary Note S1 to S8
Supplementary Figure S1 to S27
Supplementary Table S1-S6
Supplementary References

Supplementary Methods

Materials. All chemical reagents were purchased from commercial suppliers, and no further purification was required before use. Reagents including cobalt nitrate hexahydrate ($Co(NO_3)_2 \cdot 6H_2O$, Shanghai Macklin Biochemical Co., Ltd., AR, 99%), 2-methylimidazole ($C_4H_6N_2$, Aladdin., 98%), polyvinylpyrrolidone (PVP, Aladdin, molecular weight 10000), ethanol (C_2H_5O, Sinopharm Chemical Reagent Co., Ltd., AR), ammonium persulfate (APS), and acrylic acid (AA) were obtained from Sinopharm Chemicals Reagent Co., Ltd., and used without further purification. Chloroplatinic acid hexahydrate ($H_2PtCl_6 \cdot 6H_2O$, ACS reagent) was purchased from Alfa Aesar.

Characterization of the photocatalysts. Scanning electron microscopy (SEM) and energy dispersive X-ray spectroscopy (EDX) of photocatalysts were performed on an SEM (FEI Helios G4 CX 450) equipped with an EDX spectrometer. The morphology of the sample was examined by transmission electron microscopy (TEM) (FEI Talos F200X), which was equipped with EDX spectroscopy. Raman spectra were recorded by a Renishaw RM1000 laser Raman spectrometer using a laser excitation of 532 nm. X-ray diffraction (XRD) (BRUKER D2 PHASER) using Cu Kα (λ = 1.5406 Å) radiation was performed to assess the crystalline structure of the synthesized samples. The absorption properties were tested by ultraviolet-visible diffuse reflectance spectroscopy (UV–vis DRS), and the photocatalyst powders were obtained from a JASCO V-570 UV–visible/NIR spectrophotometer in diffuse absorption mode with the white standard $BaSO_4$ as a reference. The electron spin resonance (ESR) trapping measurements were collected using a JEOL JES-FA200 electron spin resonance spectrometer (298 K, 9.062 GHz). Fourier transform infrared (FTIR) spectra were recorded with a Bio-Rad FTIR spectrometer FTS165. X-ray photoelectron spectroscopy (XPS) was performed with a Kratos AXIS Ultra DLD spectrometer. Adventitious carbon (C 1 s located at 284.6 eV) was used as a reference for the binding energy. UV photoelectron spectroscopy (UPS) was measured using He I

excitation (21.2 eV) and recorded with a constant pass energy of 1 eV in the ultrahigh vacuum (UHV) chamber of the XPS instrument. The environmental relative humidity and temperature were recorded by a Center 313 Rs-232 Humidity Temperature Meter with a LabView-based data acquisition system. The temperature on the surface is measured using a multichannel temperature data recorder equipped with thermocouple sensor K type -50 °C–250 °C (Toprie TP9000).

Electrochemical measurements. Electrochemical impedance spectroscopy (EIS) measurements were acquired on an AutoLab PGSTAT204 electrochemical workstation. The frequency range was 0.1 Hz–10 kHz, and the magnitude of the modulation signal was set to be 0 V to avoid possible interference by the voltage induced. The equivalent circuit consists of an electrolyte resistance (R_E) in series connected with a combination of an electrode resistance (R_S) and a constant phase element (CPE) in parallel and serially connected with a Warburg impedance (W_O) [1-8].

Density functional theory (DFT) Calculations. First-principle calculations were performed by using the Vienna Ab-initio Simulation Packages (VASP) based on the DFT [6]. The generalized gradient approximation (GGA) of the Perdew-Burke-Ernzerhof (PBE) form was adopted for the exchange and correlation energy. The energy cutoff of plane-wave basis set was set to 400 eV. Γ-centered k-mesh with k-spacing of 0.03 $Å^{-3}$ in the Brillouin zone are used for geometry optimization and self-consistent calculation. The total energy converges within an error of 1×10^{-5} eV $atom^{-1}$ and all atoms were relaxed until the residual force was less than 0.01 eV $Å^{-1}$ during relaxation. The DFT-D2 method proposed by Grimme was applied to describe the van der Waals interactions. The CoO–NC heterostructure model was constructed by 3×4 supercell of CoO (100) surface and $2\sqrt{3}\times5$ supercell of NC surface. Partial C atoms were removed to expose the active site of CoO. A vacuum region of 15 Å was applied for eliminating interactions between the neighboring cells of slab models. The external electric field with

0.4 V was added into the CoO–NC heterostructure.

The H_2O_2 evolution process has 3 steps in neutral media, as shown in equations (1)-(3) [5, 6]:

$$* + H_2O \rightarrow *OH_2 \quad (1)$$

$$*OH_2 \rightarrow *OH + H^+ + e^- \quad (2)$$

$$*OH + H_2O \rightarrow * + H_2O_2 + H^+ + e^- \quad (3)$$

In the above equations (1)-(3), the star (*) represents the catalytic site * onto the catalyst.

The Oxygen evolution reaction (OER) process is a four-electron reaction pathway, which is described by [6]:

$$H_2O + * \rightarrow OH^* + H^+ + e^- \quad (4)$$

$$OH^* \rightarrow O^* + H^+ + e^- \quad (5)$$

$$H_2O + O^* \rightarrow OOH^* + H^+ + e^- \quad (6)$$

$$OOH^* \rightarrow * + O_2 + H^+ + e^- \quad (7)$$

where the symbol * describes an active site on the surface of a catalyst, OH^*, O^* and OOH^* show the adsorbed intermediates during oxygen evolution process.

Taking the OH* adsorption as an example, the free energy ΔG_{*OH} can be expressed as [5]:

$$\Delta G_{OH*} = \Delta E + \Delta ZPE + \Delta H - T\Delta S \quad (8)$$

where ΔE is the total energy difference for OH molecular after and before adsorbed at the substrate. ΔZPE is the zero-point energy difference, and the ΔH and ΔS are the changes of enthalpic and entropic contributions.

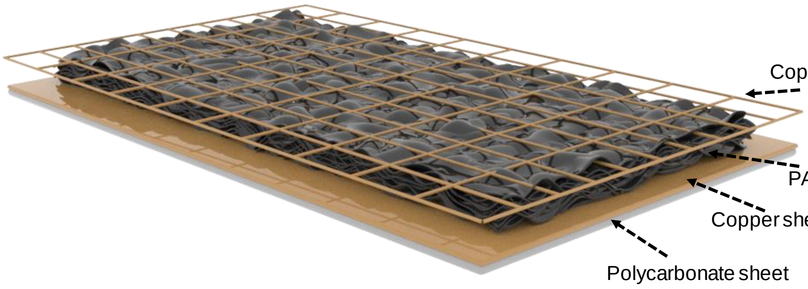

Figure S1. Device schematic and configuration used for electricity measurements.

Fabrication of devices for electric energy harvesting: to fabricate a typical device with a predesigned channel, the polycarbonate (PC) sheet as a substrate was first cut into a rectangular shape and then pasted the copper sheet as the bottom electrode. Then 50 mg of PAA/CoO–NC was dispersed in 1.5 ml of Milli-Q water to form an aqueous dispersion. Under vigorous magnetic stirring, 50 μL of ethanol solution of Span80 (5 wt%) and 5 μL of Nafion (5 wt%) solution were added into the dispersion solution. The mixture was then stirred for 2 h, yielding a paste-like dispersion solution and is introduced by drop-cast method and naturally dried to form a film. Then, the copper mesh electrode was pasted by epoxy slurry on the PAA/CoO–NC surface as the top electrode, allowing the water steam to pass through.

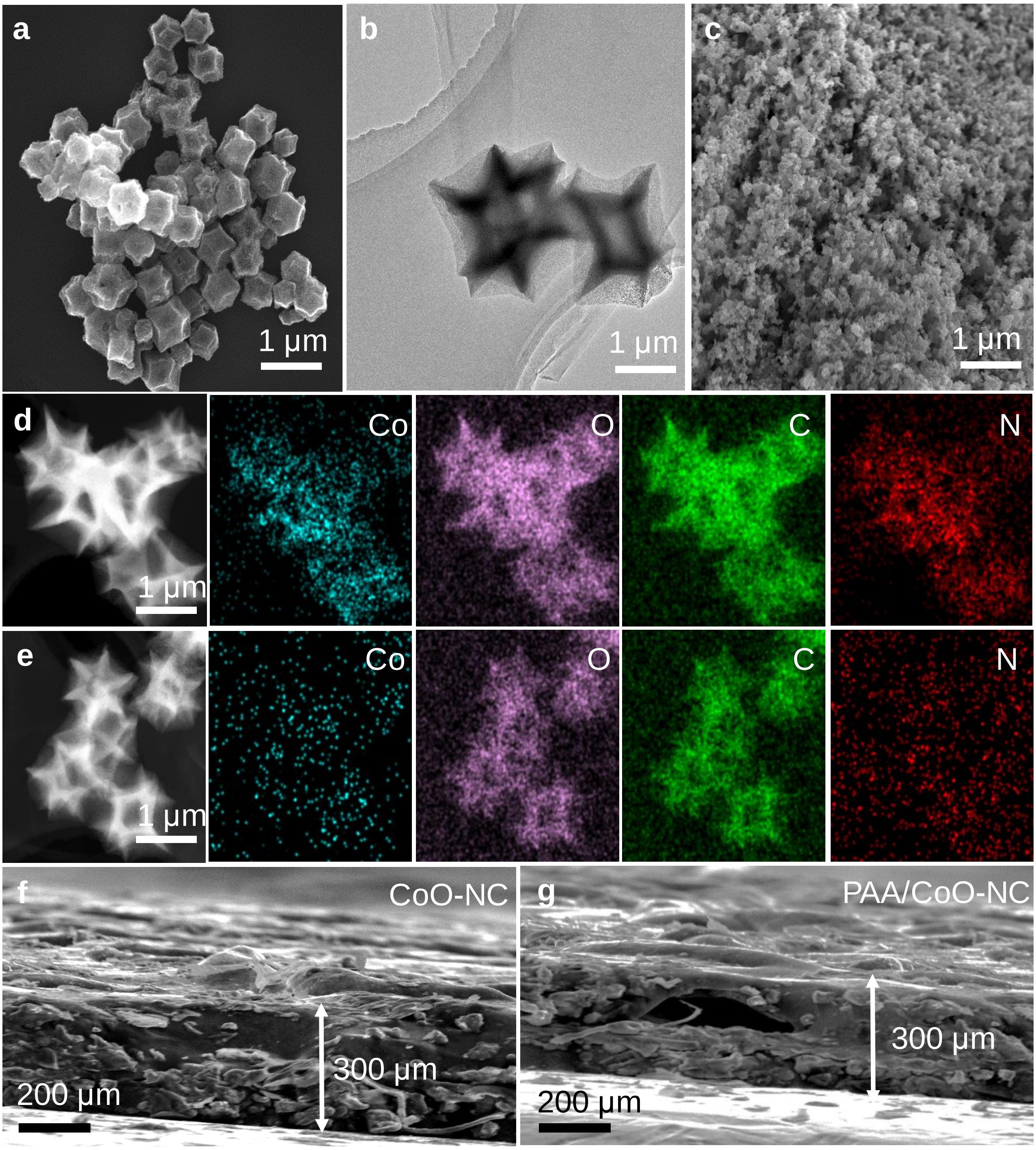

Figure S2. a SEM image of CoO–NC; **b** TEM image of CoO–NC; **c** SEM image of casting CoO–NC film; **d** EDX mapping images of CoO–NC for individual elements of Co, O, C and N, respectively; **e** EDX mapping images of PAA/CoO–NC for individual elements of Co, O, C and N, respectively; **f** Cross-sectional SEM image of CoO–NC; **g** Cross-sectional SEM image of PAA/CoO–NC. The SEM and TEM images of individual CoO–NC nanoparticles show a polyhedral morphology in Figure S2a, b. The SEM image of casting CoO–NC film shows a piled nanoparticle structure (Figure S2c). The HAADF-STEM elemental mapping images of CoO–NC show the distributions of Co, O, C and N elements (Figure S2d). The HAADF-STEM elemental mapping images of PAA/CoO–NC show the uniform distributions of Co, O, C and N elements (Figure S2e). The cross-sectional SEM images of CoO–NC and PAA/CoO–NC show the casting thicknesses of 300 µm in Figure S2f, g.

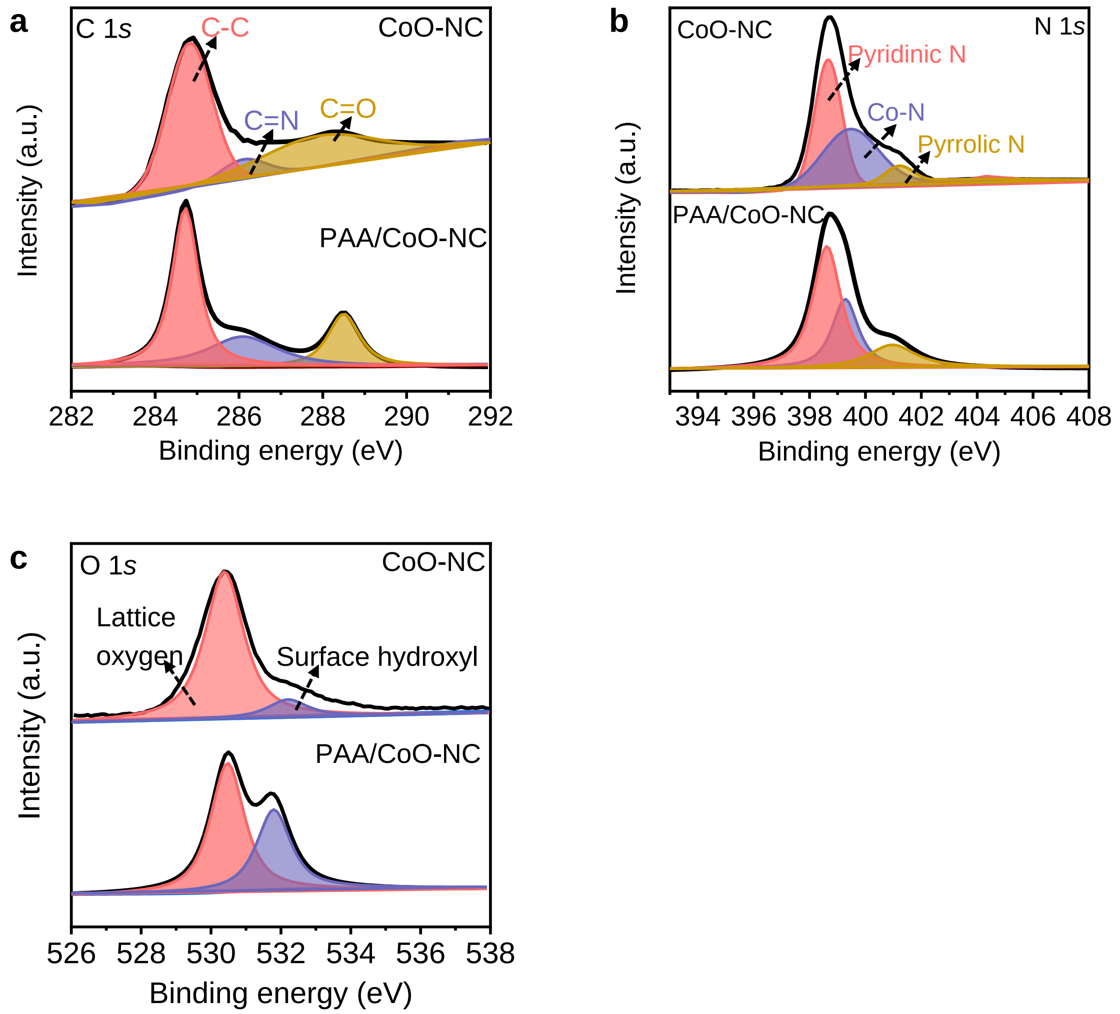

Figure S3. XPS spectra of PAA/CoO–NC and CoO–NC: **a** C 1*s* spectra; **b** N 1*s* spectra; **c** O 1*s* spectra. The C 1 *s* spectra in PAA/CoO–NC and CoO–NC show C–C, C=N, and C=O peaks at 284.6 eV, 286.3 eV and 288.5 eV, respectively, in Figure S3a [9]. The N 1 *s* spectra in PAA/CoO–NC and CoO–NC show pyridinic N, Co–N, and pyrrolic N peaks at 398.3 eV, 399.2 eV, and 401.1 eV, respectively in Figure S3b. The O 1*s* spectra can be deconvoluted into two peaks: one at 530.3 eV, which can be assigned to the lattice O in CoO. Another peak at 532.1 eV is assigned to the surface hydroxyl groups present in the samples in Figure S3c.

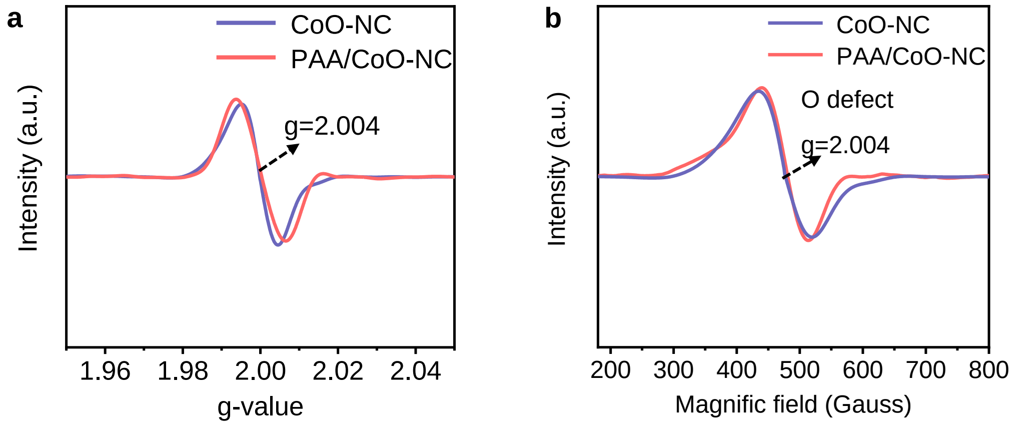

Figure S4. a, b ESR spectra of CoO–NC and PAA/CoO–NC. The ESR signal intensity at a g-value of 2.004 is related to the oxygen defect concentration in Figure S4a [9]. The ESR signal intensities demonstrate that the CoO–NC and PAA/CoO–NC possess similar concentration of oxygen defects, as shown in Figure S4b.

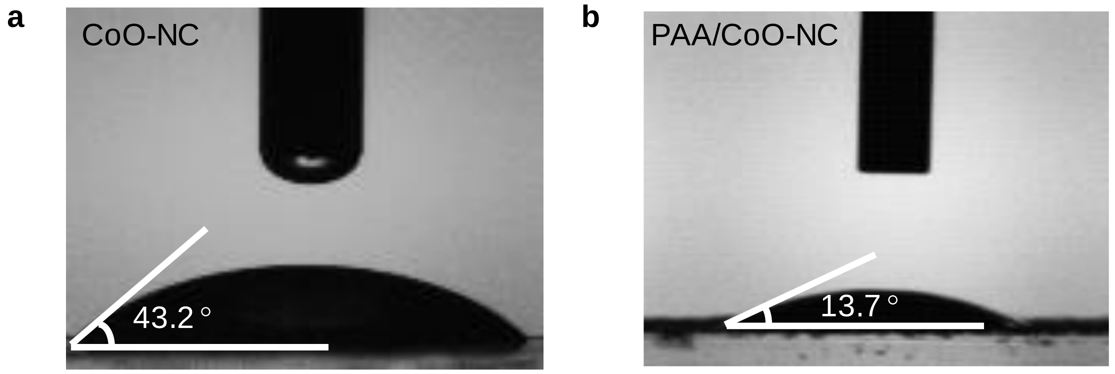

Figure S5. Images of the wetting condition of water on the **a** CoO–NC and **b** PAA/CoO–NC nanomaterials.

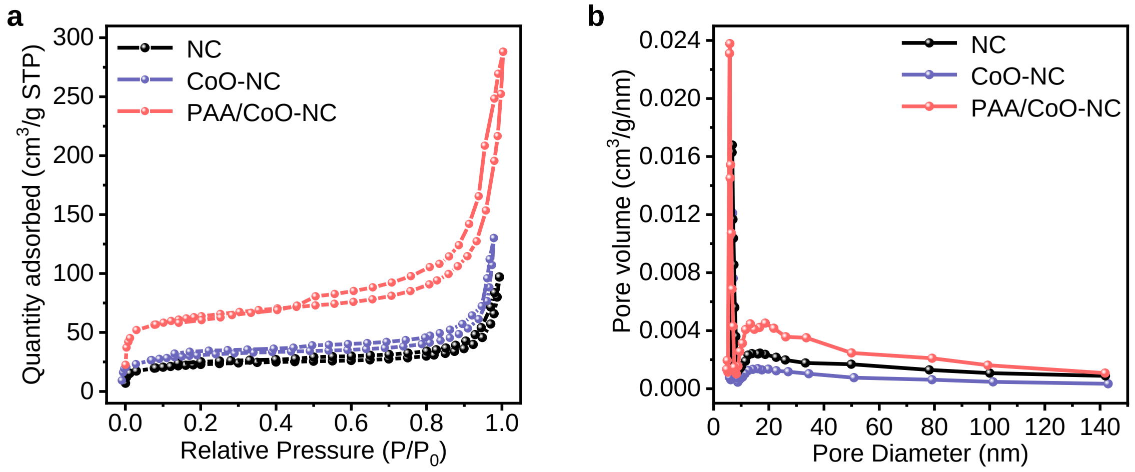

Figure S6. **a** N_2 adsorption-desorption isotherm of NC, CoO–NC and PAA/CoO–NC nanomaterials; **b** The corresponding pore size distribution curves. From the N_2 adsorption-desorption isotherm plots, the specific surface areas of PAA/CoO–NC, CoO–NC and NC nanomaterials are 102, 73, and 61 m^2 g^{-1}, respectively, as shown in Figure S6a. Moreover, the pore size distribution for PAA/CoO–NC, CoO–NC and NC nanomaterials all feature typical mesoporous characteristics as shown in Figure S6b. The combined analysis of the specific surface area and the pore

size distribution confirm the porous structures of the samples.

Note S1: Measurement of the energy harvesting process. The as-prepared PAA/CoO–NC with top and bottom electrodes was placed in an enclosed container. Moisture was applied and adjusted by flowing argon gas from water into the container [10-16]. The humidity was measured by a humidity meter (ThermoProTP50). The open-circuit voltage and short-circuit current density were tested in real time using a Keithley 2400. The circuit parameters of the open-circuit voltage test were current = 0 nA and step index = 10 points s^{-1} [17]. The circuit parameters of the short-circuit current density test were voltage = 0 mV and step index = 10 points s^{-1} [18-23]. The samples were placed in a homemade container with adjusted relative humidity by pumping a mixture of dry and wet argon gas (Ar/H_2O steam) [7].

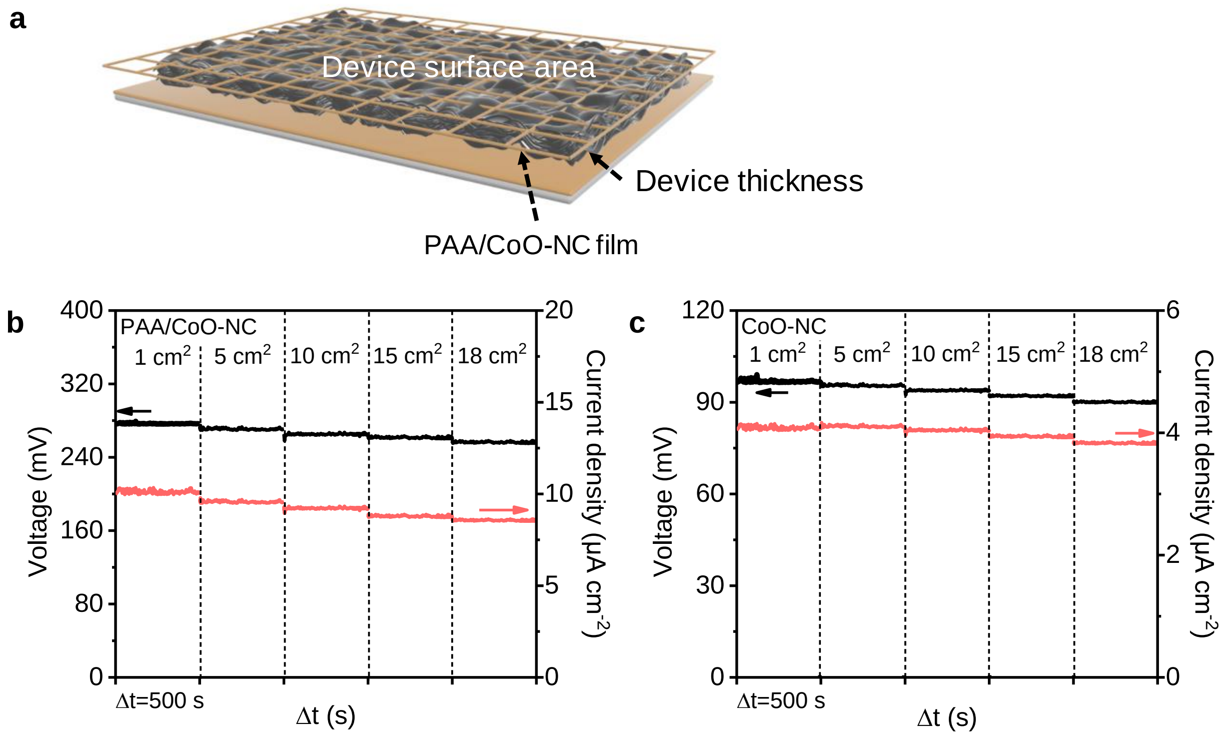

Figure S7. a The diagram of devices (surface area and thickness as important factors); **b** Measurements of voltage and current density on PAA/CoO–NC with different surface areas. Δt = 500 s. Black arrow represents voltage; Red arrow represents current density.

The PAA/CoO–NC and CoO–NC devices with sandwiched electrodes for electricity tests are shown in Figure S7a. As the surface area of the device increased from 1 cm^2 to 18 cm^2, the voltage and current density for PAA/CoO–NC and CoO–NC all showed a slight decrease, which is due to the

additional defects introduced into the devices as the surface area increases. The voltages are measured to be ≈ 280 and ≈ 100 mV for PAA/CoO–NC and CoO–NC devices, respectively, while the current density showed a basically stable value around ≈ 12 and ≈ 4 μA cm^{-2} for PAA/CoO–NC and CoO–NC device, respectively (Figure S7b, c). These results indicate that the surface area of device has little effect on the electricity generation for the devices. Considering a larger surface area of the device is beneficial for photocatalysis due to complete exposure to light, we selected the device surface area of 15 cm^2 for the generation of electricity and its photocatalytic reactions.

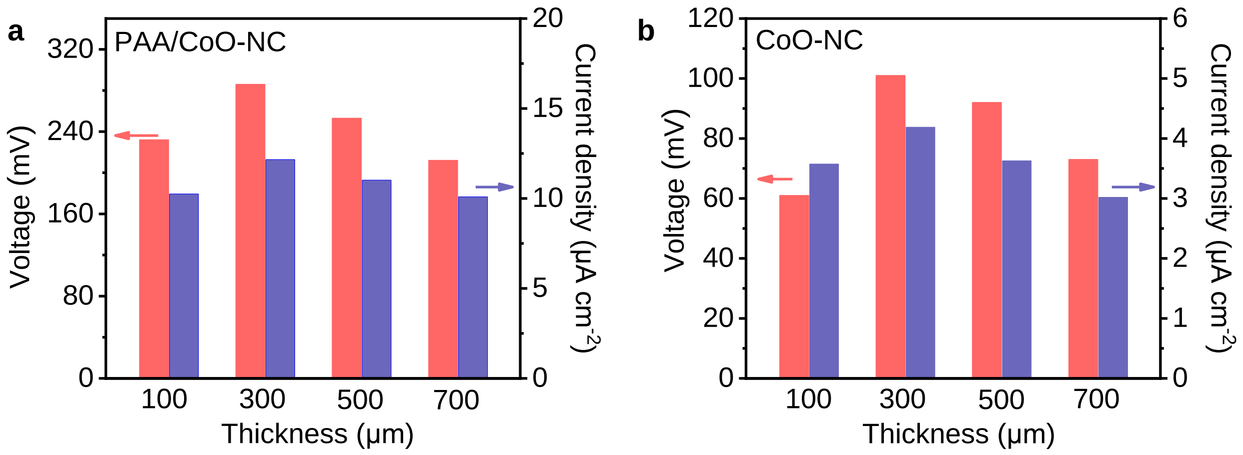

Figure S8. a The effect of different thicknesses on the voltage and current density measurements for PAA/CoO–NC in a closed reactor with 1100 ml h^{-1} Ar/H_2O steam flow (area of 15 cm^2); **b** The effect of different thicknesses on the voltage and current density measurements for CoO–NC in a closed reactor with 1100 ml h^{-1} Ar/H_2O steam flow (area of 15 cm^2). Red arrow represents voltage; Blue arrow represents current density.

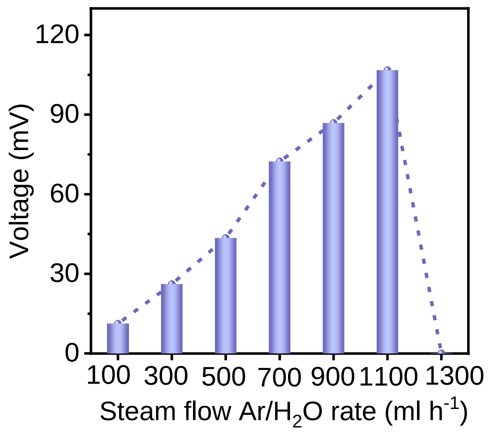

Figure S9. The output voltage of CoO–NC with different Ar/H_2O mixture flow rates of 100, 300, 500, 700, 900, 1100 and 1300 ml h^{-1}.

As the electrical output derives from the interaction between moisture and the system, the RH in the nanomaterials plays a key role in the performance. The nonlinear relationship between voltage (or current) and RH may be ascribed to the percentage of ions in a fixed amount of water increasing with RH [22, 23]. The voltage is generated from the diffusive flow of ions in the water. A high concentration of ions can build a large gradient of ions, which improves the diffusion of ions along the channels and results in a large electricity output. The positive ions in water mainly come from the weak ionization between water molecules, a reaction generating both H^+ and OH^- ($H_2O \leftrightarrow H^+ + OH^-$). For a given amount of water molecules with low RH, the amount of ions will decrease because of the low probability of ionization reaction due to the larger average distances between water molecules. The CoO–NC reaches the highest voltage of 106 mV at a 1100 ml h^{-1} rate of Ar/H_2O, suggesting that a strong correlation exists between the electrical generation performance and wetting behavior.

Note S2: Factors of voltage tuning for light illumination on PAA/CoO–NC. The voltage output is a balance of free-proton transport driven by the water steam concentration difference. System temperature has been elevated by light illumination. The relationship can be mathematically described as the following diffusion coefficient equations of (9) and (10) [16, 24, 25]:

$$D = \mathrm{D}_0\, e^{E_a} / \mathrm{R}T \tag{9}$$

$$D\, e \frac{\partial n}{\partial x} = \sigma \frac{\partial U}{\partial x} \tag{10}$$

where T, R, D, D_0, E_a, e, $\frac{\partial n}{\partial x}$, σ, and $\frac{\partial U}{\partial x}$ denote the temperature, gas constant, diffusion coefficient of protons in adsorbed water layer, maximum diffusion coefficient at infinite temperature, the energy that activates the diffusion coefficient, the unit electric charge, the concentration gradient of protons, the electrical conductivity of the adsorben water layer, and the voltage drop gradient along the device.

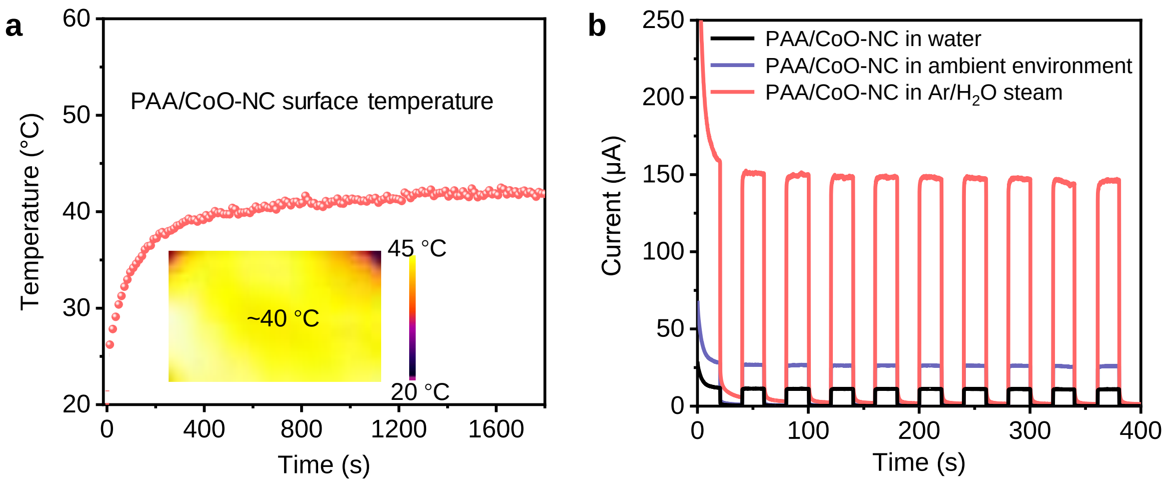

Figure S10. a The detected temperature on PAA/CoO–NC surface over time with light illumination (light intensity: AM 1.5G, 100 mW cm^{-2}) and at Ar/H_2O injection rate of 1100 ml h^{-1}; **b** Measured I-t characteristics of PAA/CoO–NC film for different testing conditions.

The light illumination induced an elavated temperature of PAA/CoO–NC surface of about 40 °C, as revealed by Figure S10a. As the temperature increases of the surface, the thermal motion of molecules and the collisions between molecules are intensified, and the activation energy E_a is greatly increased, so is the diffusion coefficient D, thus increasing the output voltage according to equation (9) and (10). As a result, the light illumination induces a higher temperature on the surface of

PAA/CoO–NC, bringing an increased diffusion and moving velocity of water steam in the nanochannel of PAA/CoO–NC.

In addition to an increased velocity of water steam induced by the light illumination contributes to an increased voltage output. The inhomogeneous distribution of the heat (i.e. thermo-electric effect) and photogenerated carriers (i.e. photoelectric effect) induced by light illumination have effects on an enhanced voltage generation. As shown in Figure S10b, the photocurrent measurement of PAA/CoO–NC film was taken to detect the photogenerated current by using Keithly 2400 source meter under three testing conditions of the device. The three conditions are tested below. (i) The PAA/CoO–NC was tested in water to avoid hydrovoltaic effect and thermo-electric effect. The obtained transient current is supposed to be the photoelectric effect induced photocurrent. As soon as the light is turned on, the transient current induced by the photogenerated increases instantaneously to about 8 μA, indicating the photogenerated carriers' motion does exist in the system, which affects the voltage output; (ii) The PAA/CoO–NC was tested in the ambient environment without water steam introduction to retaining its thermo-electric effect induced by the inhomogeneous distribution of the heat, and photoelectric effect induced by photogenerated carriers. The transient current increases to about 25 μA with light illumination, indicating the thermo-electric effect exists in the system under light illumination; (iii) The PAA/CoO–NC was tested in the water steam environment with light illumination in case of the presence of photoelectric, thermo-electric, and hydrovoltaic effects. The transient current showed a maximum of about 150 μA, indicating an enhanced effect induced by hydrovoltaic effect. To sum up, the light illumination on the surface of PAA/CoO–NC induced an elevated temperature on the surface and resulted in the moving velocity of water steam (hydrovoltaic effect), photogenerated carriers' motion, and thermo-electric effect, which collectively contributed to a higher output voltage.

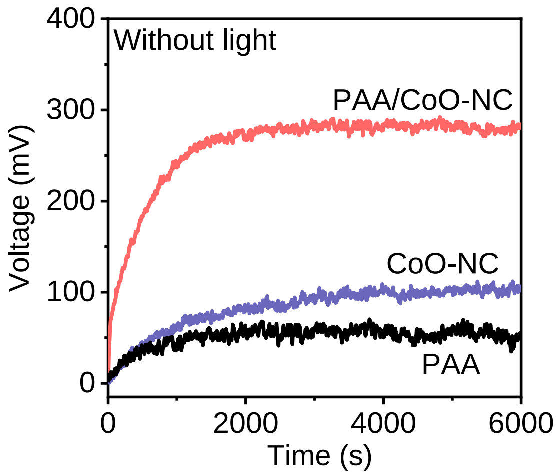

Figure S11. The measured output voltage for the CoO–NC, PAA and PAA/CoO–NC over time without light illumination. The samples perform lower voltages without light illumination. The PAA/CoO–NC, PAA, and CoO–NC exhibit voltages of 287 mV, 58 mV and 103 mV, respectively.

Note S3: Calculation of the maximum power output of devices. In a device with a channel size of 3 cm (L) x 5 cm (W), V_{oc} and I_{sc} can be found in Figure 2 in the main text, which are 402 mV and 16.9 μA cm^{-2}, respectively. Therefore, the maximum output power (P_{max}) can be estimated using the following equation (11) [10]:

$$\begin{aligned} P_{max} &= \frac{1}{4} \times V_{oc} \times I_{sc} \times \text{Width} \times \text{Length} \\ &= \frac{1}{4} \times 402\ \text{mV} \times 16.9\ \mu\text{A cm}^{-2} \times 5\ \text{cm} \times 3\ \text{cm} \\ &\approx 25.4\ \mu\text{W} \end{aligned} \tag{11}$$

Therefore, the corresponding maximum power density can be calculated based on the volume of PAA/CoO–NC film or the mass of the PAA/CoO–NC film. The typical thickness of the PAA/CoO–NC film is measured to be approximately 300 μm. Therefore, the maximum power density against film volume is estimated using the following equation (12) [11]:

$$\begin{aligned} \text{Power density}_{\text{volume}} &= \frac{P_{max}}{\text{volume}} \\ &= \frac{25.4\ \mu\text{W}}{3\ \text{cm} \times 5\ \text{cm} \times 300 \times 10^{-3}\ \text{cm}} \\ &= 5.6\ \mu\text{W cm}^{-3} \end{aligned} \tag{12}$$

The maximum power density against mass is calculated based on the amount of PAA/CoO–NC used to cast the film (50 mg) and estimated using the following equation (13) [12]:

$$\begin{aligned} \text{Power density}_{\text{mass}} &= \frac{P_{max}}{\text{mass}} \\ &= \frac{25.4\ \mu\text{W}}{5 \times 10^{-2}\ \text{g}} \\ &= 509.5\ \mu\text{W g}^{-1} \end{aligned} \tag{13}$$

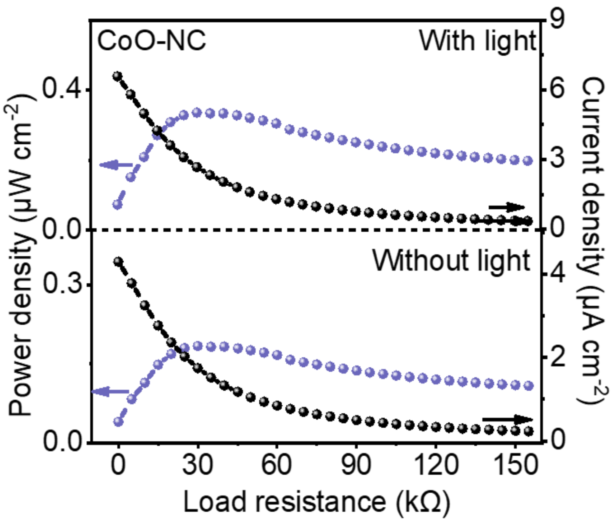

Figure S12. Output power and current density of CoO–NC under different load resistances with or without light illumination. The power performance for the CoO–NC was further investigated by connecting different external load resistances ranging from 1 Ω to 150 kΩ. At a load resistance of 33 kΩ, the power density reaches a maximum of 0.33 μW cm^{-2} with light illumination and 0.19 μW cm^{-2} without light illumination, while the current density gradually drops to zero. Blue arrow represents power density; Black arrow represents current density.

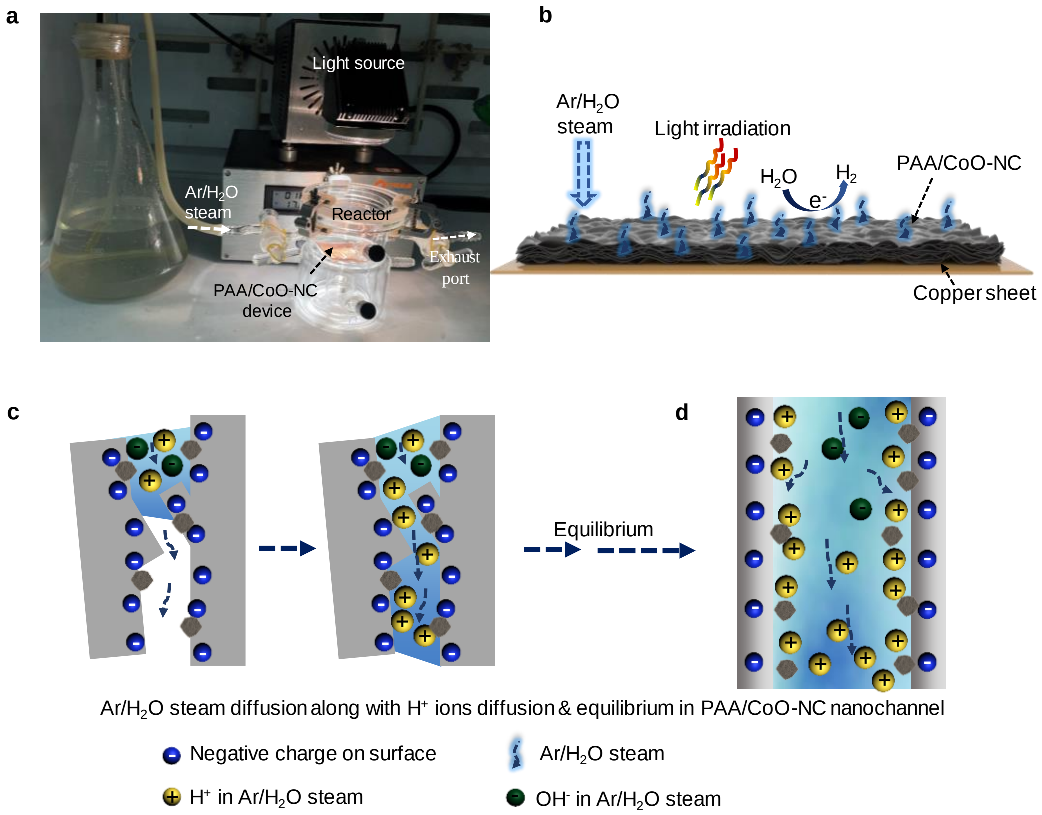

Figure S13. a Photo of the PAA/CoO–NC hydroelectric generator for electricity generation under light illumination (AM 1.5G, 100 mW cm^{-2}) with Ar/H_2O injection; **b** The schematic of Ar/H_2O steam diffusion from the PAA/CoO–NC surface to the bottom of the film; **c** Schematic illustration of classical electrokinetic effect in the nanochannel of PAA/CoO–NC with Ar/H_2O steam flow and the ions diffusion along the negatively charged walls of PAA/CoO–NC nanochannels; **d** Equilibrium of Ar/H_2O steam and ions diffusion in a nanochannel of PAA/CoO–NC.

Figure S13a shows the photo of PAA/CoO–NC hydroelectric generator. The PAA/CoO–NC is placed in a closed reactor, where the wetted Ar gas (Ar/H_2O) is brought into, and a certain ambient humidity is formed in the reactor by controlling the Ar gas rates. Figure S13b shows the schematic of water steam diffusion on PAA/CoO–NC film. Generally, when water molecules are exposed on the surface of PAA/CoO–NC film, the water molecules enable mobility of H^+ ions along water chains from the surface deep into the nanomaterials network. The diffusion direction of water steam is perpendicular to the membrane downward, which is similar to the understanding of moisture diffusion in membrane of pores in the previous literature [25]. The hydrovoltaic voltage is generated because of a gradient in the concentration of water molecules along the nanochannels from the top surface to the

bottom of film as illustrated in Figure S13c, d. The moisture of Ar/H_2O, acting as an external force, affects the diffusion of water, which in turn creates the relative motion of water molecules and solids of PAA/CoO–NC. The PAA/CoO–NC generates the double layer overlaps and a pressure-driven flow carriers counter-ions of H^+ to form an electric current in the flow, eventually reaching an equilibrium of H^+ ions diffusion, resulting in a constant electrokinetic voltage.

Note S4: Theoretical modelling and finite element method simulations. The ionic migration-induced electricity generation mechanism of a moist electric generator was analysed by a theoretical model of the Gouy–Chapman model based on the Nernst–Plank and Poisson equations (NPP) with proper boundary conditions to elucidate the underlying mechanisms of charge transfer and storage, as well as ion diffusion [16, 23].

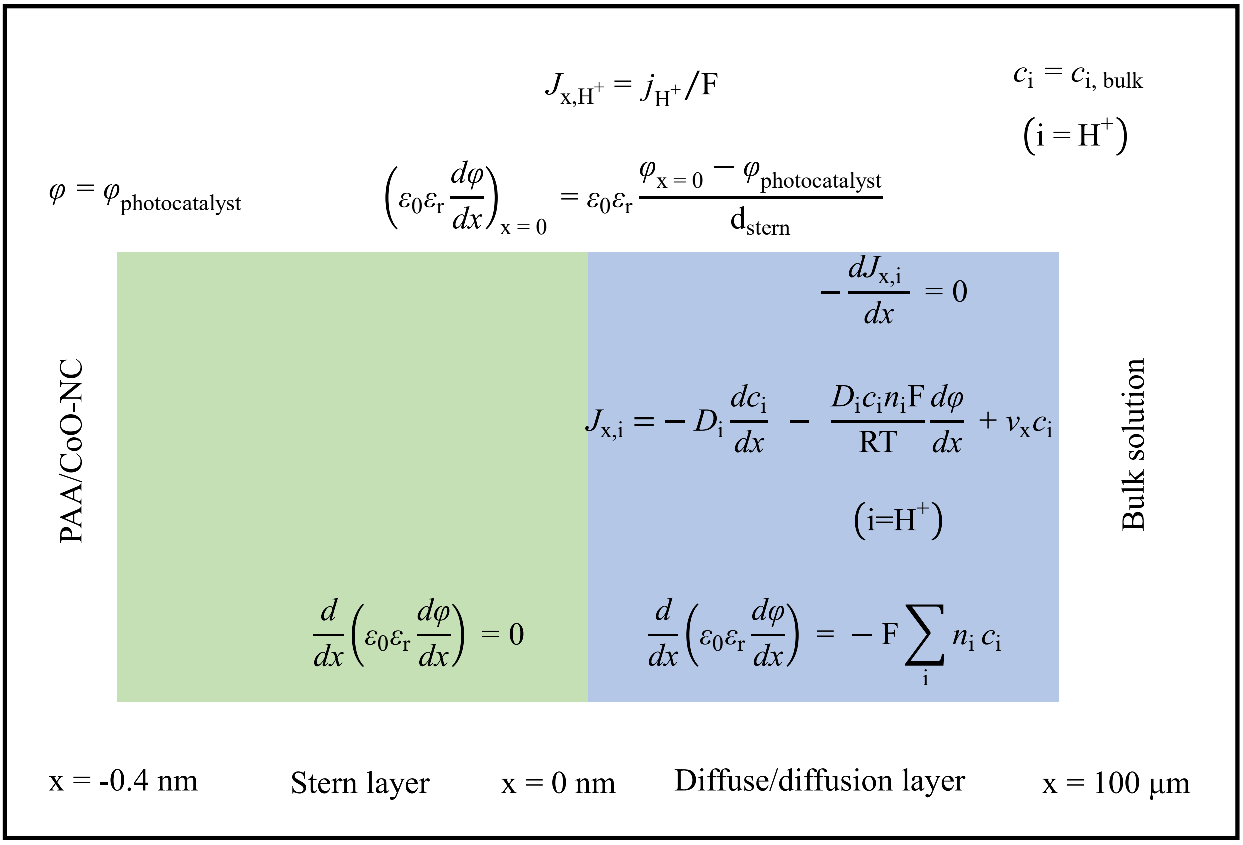

The Poisson equation is described by [23]:

$$\nabla\cdot(\varepsilon_0\varepsilon_r\nabla\varphi) = -\sum i\, z_i\, e\, C_i \tag{14}$$

$$\nabla^2\varphi = -\frac{F}{\varepsilon}\sum z_i\, C_i \tag{15}$$

and the Nerst–Plank equation [23]:

$$\frac{\partial C_i}{\partial t} = \nabla\cdot\left[D_i\, \nabla\, C_i + \frac{D_i C_i}{K_B T}\, z_i\, e\, \nabla\varphi\right] \tag{16}$$

$$j_i = D_i\left(\nabla C_i + \frac{z_i F C_i}{RT}\nabla\varphi\right) \tag{17}$$

$$\Delta\cdot j_i = 0 \tag{18}$$

where φ, F, ε, z, c, D, j, R and T are the electric potential, Faraday constant, dielectric constant, valence of ionic species, ion concentration, diffusion coefficient, ionic flux, ideal gas constant and temperature, respectively. ε_0 is the permittivity of vacuum, ε_r is the relative permittivity of the medium, D_i is the diffusivity of chemical species i, C_i is the density of the species, z_i is the valency of the species, e is the elementary charge, k_B is Boltzmann's constant, and T is the temperature.

We used the "Electrostatica" and "Transport of Diluted Species" physics of COMSOL 5.5 to obtain the electrochemical behavior of all species. The mean pore diameter of PAA/CoO–NC is approximately 20 nm. The thickness of the Helmholtz layer is taken as the radius of hydrated water and hydronium ions (0.27 nm), and the absolute temperature T is taken as 297.3 K. The dielectric constant of PAA/CoO–NC is estimated by adopting the ratio of CoO–NC and PAA with a linear relationship and is heavily correlated with the relative humidity. The diffusion coefficients for carriers in the solid electrolyte are assumed to be 1.0 x 10^{-15} m^2 s^{-1}. The ion concentration of the movable carrier is assumed to be 2.2 x 10^{-8} mol L^{-1} and changes with different relative humidity. The boundary condition for the induced potential on the nanoframe membrane surface is described by [23]:

$$\vec{\mathbf{n}} \cdot \nabla\varphi = -\frac{\sigma}{\varepsilon} \tag{19}$$

where σ is the surface charge density that depends on the ionic concentration. The ion flux in the steady state has zero normal components at the boundaries [23]:

$$\vec{\mathbf{n}} \cdot j = 0 \tag{20}$$

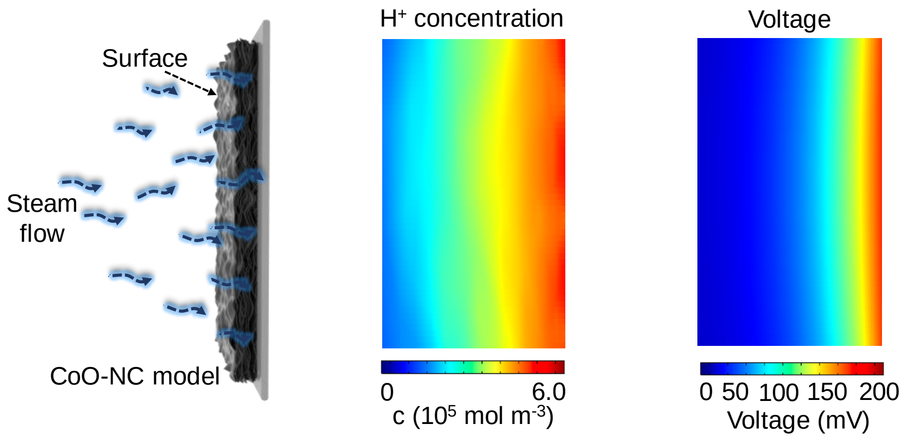

Figure S14. Numerical simulations of the electric field and surface H^+ ion distributions on the CoO–NC in cross sectional view. The simulated voltage for CoO–NC is optimally up to 200 mV, close to the experimental value. The H^+ ion distribution varies across the porous structure of the material and shows a jump from the upper surface to the bottom on which water flows.

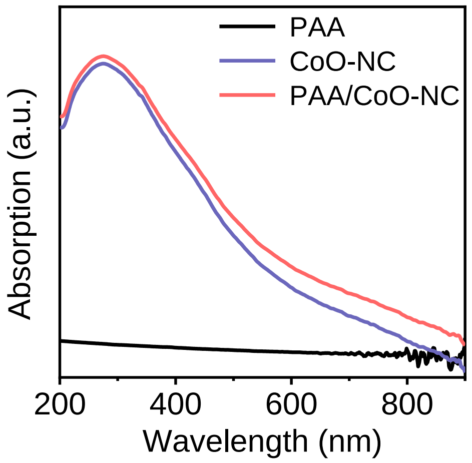

Figure S15. UV–vis DRS spectra of PAA, CoO–NC, and PAA/CoO–NC nanomaterials. PAA has no optical absorption property, showing a flat absorption intensity of almost zero. The UV–vis DRS spectra of PAA/CoO–NC and CoO–NC exhibit similar optical absorption up to the visible region, showing beneficial light absorption for photocatalytic reactions.

Note S5: Determination of the H_2O_2 concentration. The concentration of the generated H_2O_2 was determined through a titration process. After photocatalysis, the as-produced H_2O_2 solution was collected and evaluated using the standard potassium permanganate (0.1 M $KMnO_4$ solution, Sigma-Aldrich) titration process, according to the following equation [26]:

$$2MnO_4^- + 5H_2O_2 + 6H^+ \rightarrow 2Mn^{2+} + 5O_2 + 8H_2O \quad (21)$$

Sulfuric acid (1 M H_2SO_4) was used as the H^+ source.

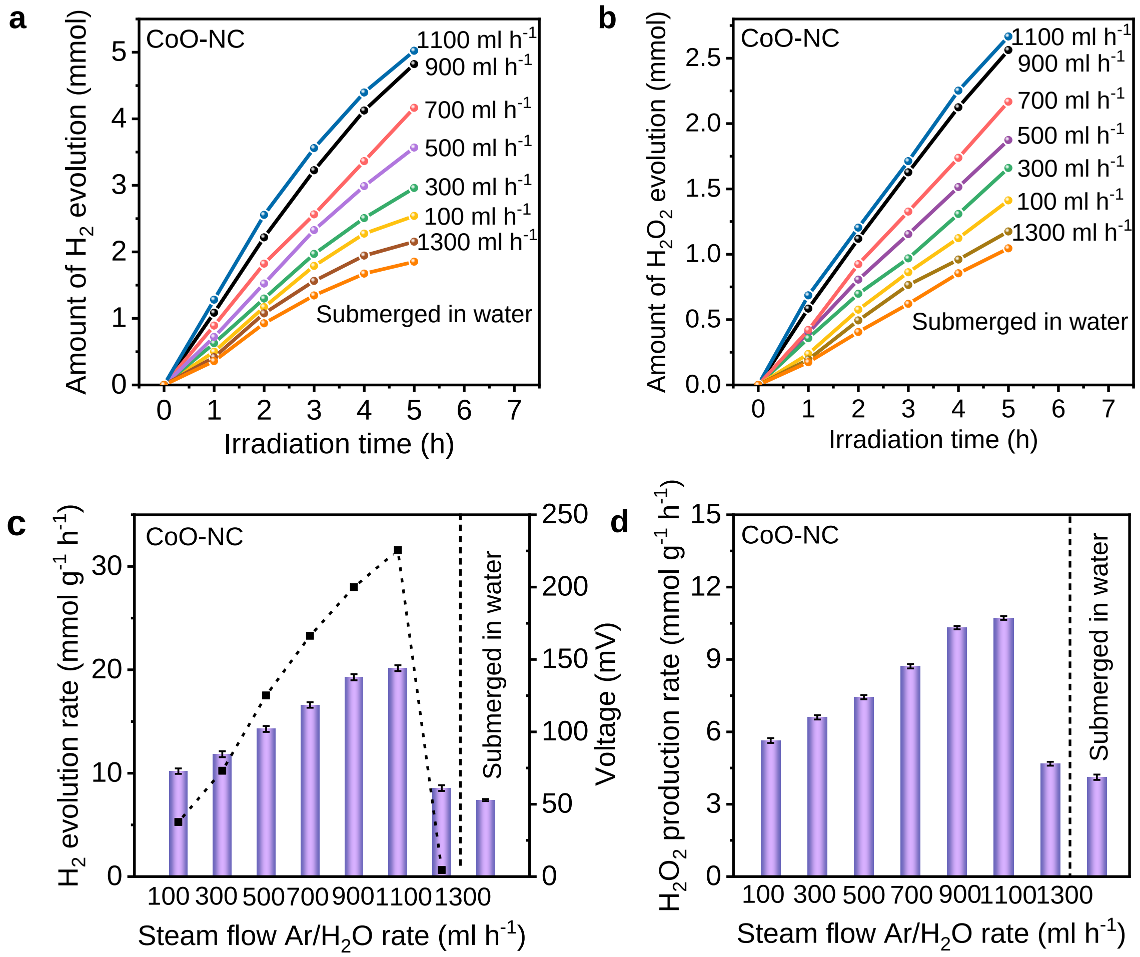

Figure S16. a Time-dependent photocatalytic hydrogen production of with different mixture gas (Ar/H_2O) flow rates of 100, 300, 500, 700, 900, 1100 and 1300 ml h^{-1}, and submerged in water; **b** Time-dependent photocatalytic H_2O_2 production of CoO–NC with different steam (Ar/H_2O) flow rates of 100, 300, 500, 700, 900, 1100 and 1300 ml h^{-1} and submerged in water; **c** The photocatalytic H_2 production rate of CoO–NC with different steam flow rates of 100, 300, 500, 700, 900, 1100 and 1300 ml h^{-1}, according to the voltage change, and submerged in water. Error bars represent the standard deviations from the statistic results of three sets of experiments; **d** The photocatalytic H_2O_2 production rate of CoO–NC with different flow rates of 100, 300, 500, 700, 900, 1100 and 1300 ml h^{-1} and submerged in water. Error bars represent the standard deviations from the statistic results of three sets of experiments. For the photocatalytic water splitting reaction, Pt cocatalyst is loaded through a photodeposition method; The light source is a solar simulator at AM 1.5G illumination (100 mW cm^{-2}).

The photocatalytic performance was investigated at different rates of Ar/H_2O injection (from 100 to 1300 ml h^{-1}), and no sacrificial agent was added. Different Ar/H_2O injection rates vary the ambient humidity of the reactor, and the H_2 production of CoO–NC gradually improves from 2.5 mmol to 5.0 mmol in five hours as the Ar/H_2O injection rate increases from 100 ml h^{-1} to 1100 ml h^{-1} (Figure S16a). The photocatalytic performance trend is consistent with the variation in the hydrovoltaic electric field,

as shown in Figure S16c. The H_2 production drops to 2.1 mmol due to a vanished hydrovoltaic effect at an Ar/H_2O rate of 1300 ml h^{-1}, comparable to that submerged in water of 1.8 mmol. The main oxidation product is H_2O_2, and no oxygen is detected in CoO–NC during the photocatalytic process. The H_2O_2 production amount for CoO–NC increases from 1.4 mmol to 2.7 mmol with increasing hydrovoltaic electric field, which has a similar trend of H_2 production in Figure S16b. The corresponding H_2O_2 production rate for CoO–NC is shown in Figure S16d.

Note S6: Calculation of the apparent quantum efficiency (AQY). The H_2 generation tests were conducted in the PAA/CoO–NC generator using a 300 W Xenon lamp irradiation with $\lambda > 300$ nm (PLS-SXE300, Beijing Perfectlight Technology Co., Ltd, 300 mW cm^{-2}). The dependence of different wavelengths for H_2 generation and AQY calculations by various band-pass filters in PAA/CoO–NC system were shown in Table S4. The apparent quantum efficiency (AQY) was measured under irradiation with monochromatic light with different wavelengths of 400, 420, 425, 450, 500, 550, and 650 nm, which was calculated from the following equation [28-30]:

$$\mathrm{AQY} = \frac{2 \times \text{the number of evolved } \mathrm{H_2} \text{ molecules}}{\text{the number of incident photons}} \times 100\%$$

$$= \frac{2 \times n_{H_2} \times \mathrm{N_A} \times \mathrm{h} \times \mathrm{c}}{I \times S \times t \times \lambda} \tag{22}$$

where n_{H_2} is the amount of H_2 molecules, $\mathrm{N_A}$ is Avogadro's constant, h is the Planck constant, c is the speed of light, I is the intensity of irradiation light, S is the irradiation area, t is the photocatalytic reaction time, and λ is the wavelength of the monochromatic light.

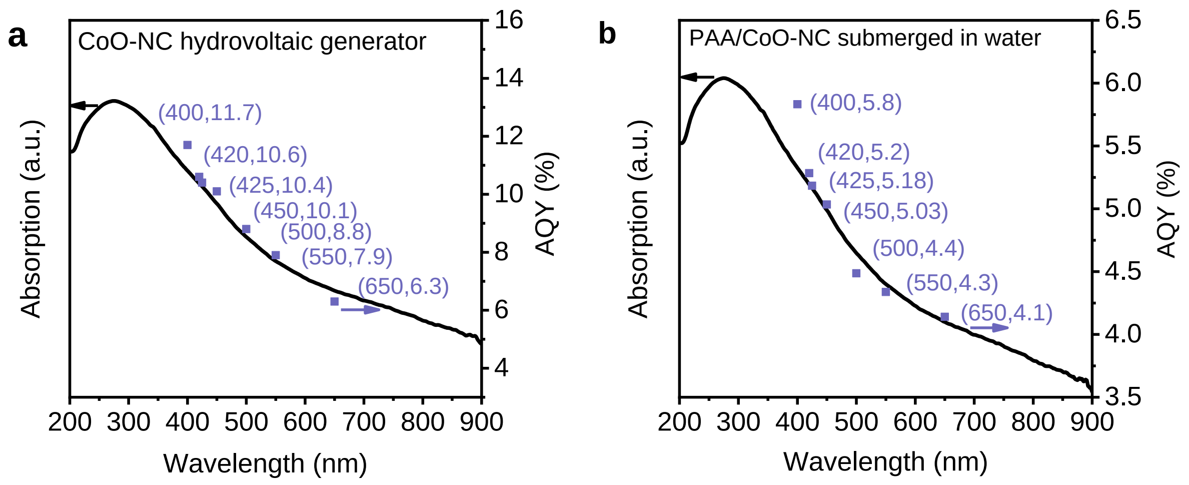

Figure S17. a UV–vis DRS spectra and wavelength-dependent AQY of CoO–NC for H_2 production; **b** UV–vis DRS spectra and wavelength-dependent AQY of PAA/CoO–NC submerged in water for H_2 production. The AQY for H_2 evolution was measured with various bandpass filters to provide monochromatic light at an Ar/H_2O rate of 1100 ml h^{-1}. The AQY at 400 nm is measured to be 11.7% and 5.8% for the CoO–NC hydrovoltaic generator and PAA/CoO–NC submerged in water without hydrovoltaic effect, respectively. Blue arrow represents AQY; Black arrow represents Absorption.

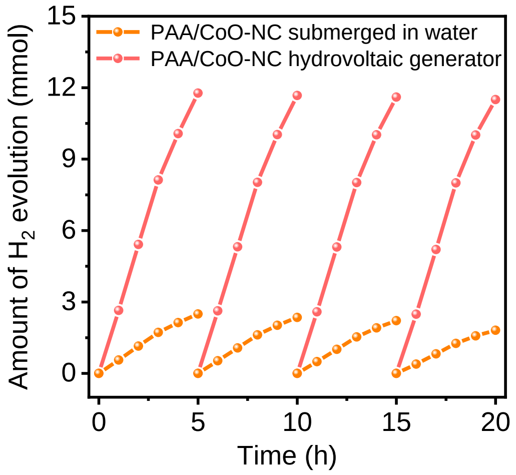

Figure S18. Cycling of photocatalytic hydrogen evolution over PAA/CoO–NC hydrovoltaic generator and PAA/CoO–NC submerged in water; The mass of photocatalyst is 50 mg. No noticeable photocatalytic activity degradation for PAA/CoO–NC hydrovoltaic generator is observed after four cycles of 20 h, while the photocatalytic performance of the PAA/CoO–NC submerged in water for testing degrades to 84% of its initial performance, indicating the PAA/CoO–NC hydrovoltaic generator is a stable system for photocatalytic water splitting.

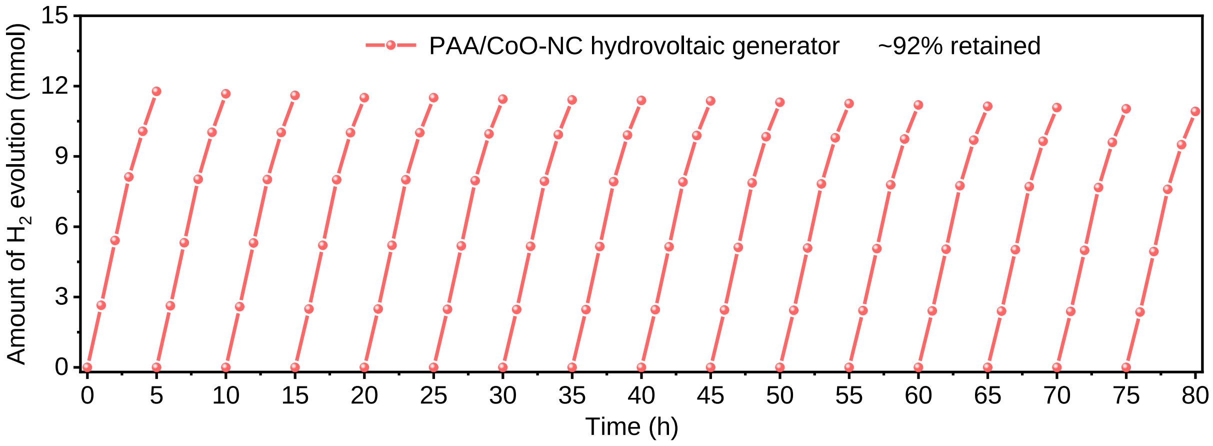

Figure S19. Cycling of photocatalytic hydrogen evolution over PAA/CoO–NC hydrovoltaic generator for a reaction period of 80 h.

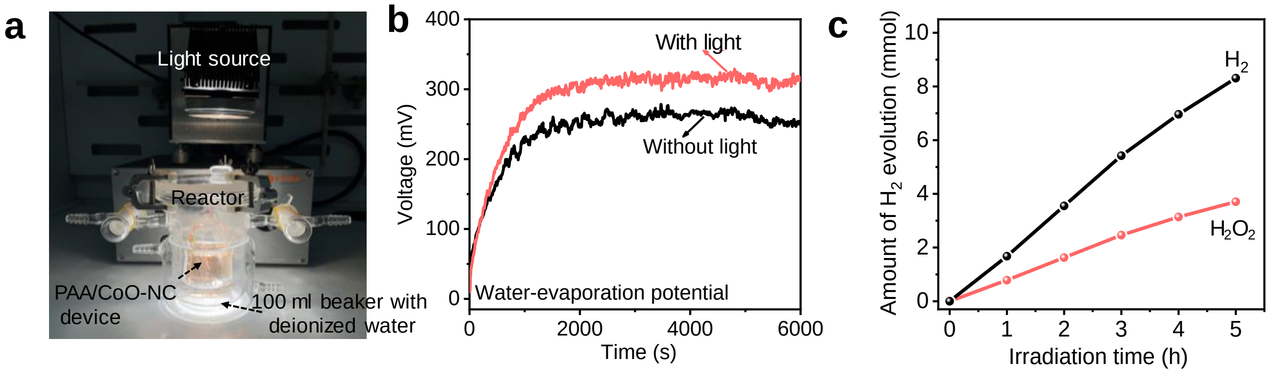

Figure S20. a Photo of the PAA/CoO–NC hydroelectric generator for measuring water evaporation-induced voltage under light illumination (AM 1.5G, 100 mW cm^{-2}); **b** Measured output voltage for PAA/CoO–NC over time with or without light illumination (light intensity: AM 1.5G; 100 mW cm^{-2}); **c** Time-dependent photocatalytic H_2 and H_2O_2 production of PAA/CoO–NC; Pt cocatalyst is loaded using a photodeposition method; The light source is a solar simulator at AM 1.5G illumination (100 mW cm^{-2}).

Figure S20a shows the photo of PAA/CoO–NC film for water-evaporation hydrovoltaic generation. The PAA/CoO–NC generator was inserted into a 100 ml beaker with deionized water covering the bottom end electrode under light illumination. As shown in Figure S20b, an open-circuit voltage between the two electrodes is generated and gradually rises to 267 mV when the capillary water reaches its maximum height along the PAA/CoO–NC sheet in about 30 mins. The voltage output increases to 318 mV with light illumination at AM 1.5G illumination (100 mW cm^{-2}). As shown in Figure S20c, the H_2 evolution amount of PAA/CoO–NC nanogenerator is 8.3 mmol, and the oxidation product of H_2O_2 is measured to be 3.7 mmol, suggesting an enhanced photocatalytic performance by the water-evaporation-induced hydrovoltaic effect.

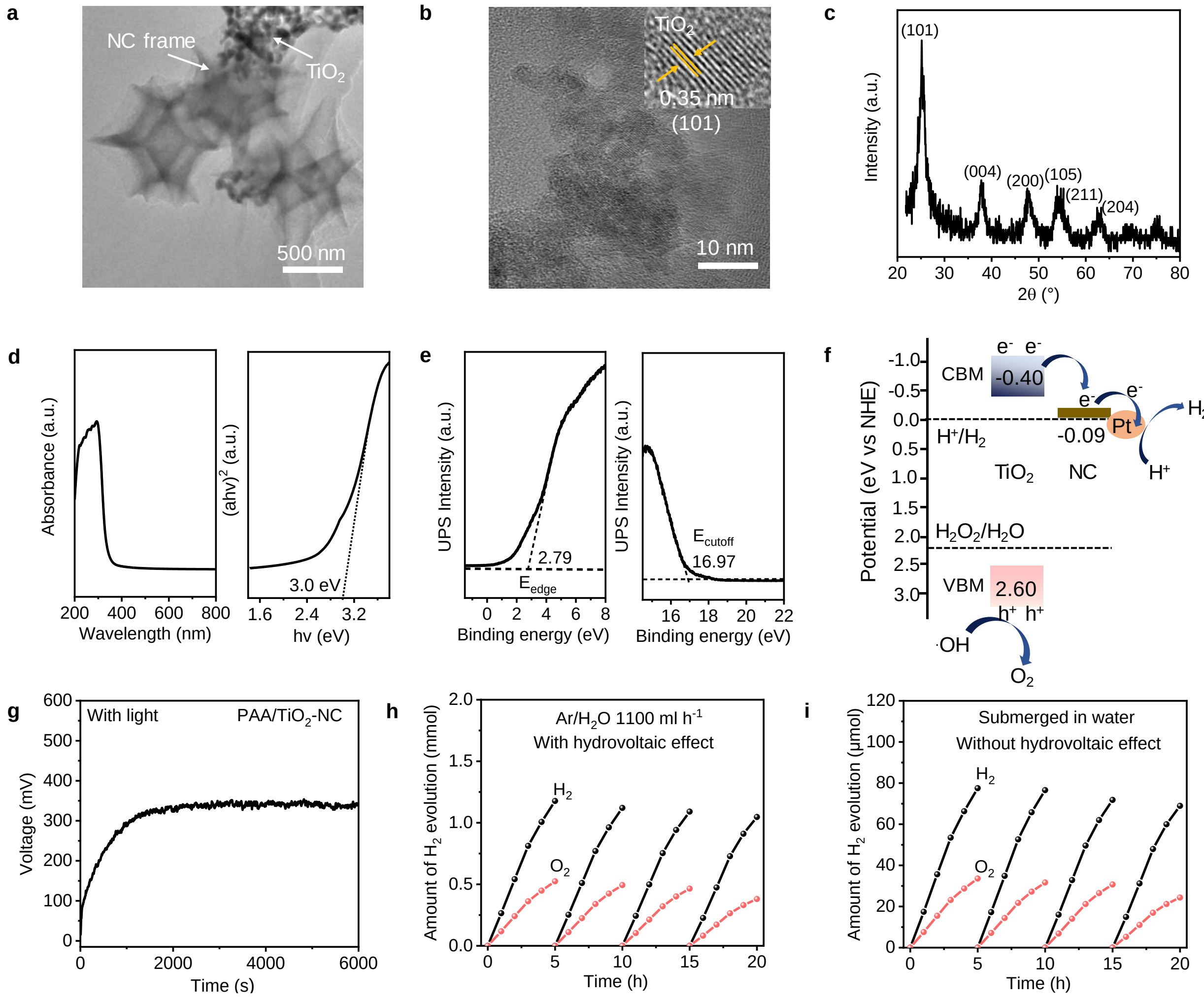

Figure S21. a TEM image of PAA/TiO_2–NC; **b** HRTEM image of PAA/TiO_2–NC; **c** XRD pattern of PAA/TiO_2–NC; **d** UV–vis DRS spectra and Kubelka-Munk function vs. the energy of incident light plots for TiO_2; **e** UPS spectrum of the valence band region of TiO_2 and UPS spectra of the cutoff region for TiO_2; **f** The corresponding energy band structures for the materials; **g** Measured output voltage for PAA/TiO_2–NC over time with light illumination (light intensity: AM 1.5G; 100 mW cm^{-2}); **h** Time-dependent photocatalytic H_2 and O_2 production of PAA/TiO_2–NC at different Ar/H_2O rate at 1100 ml h^{-1}, and submerged in water; Pt cocatalyst is loaded using a photodeposition method; The light source is a solar simulator at AM 1.5G illumination (100 mW cm^{-2}); **i** The corresponding time-dependent photocatalytic H_2 and O_2 production submerged in water.

As shown in Figure S21, NC frame and TiO_2 nanoparticles were synthesized by heat treatment and hydrothermal method, respectively, and then the two materials were then ultrasoundingly combined and crosslinked by PAA to form PAA/TiO_2–NC. The TEM images and XRD pattern have been conducted to confirm the structures of PAA/TiO_2–NC [27] (Figure S21a-c). The energy band structure of PAA/TiO_2–NC based on UV–vis DRS and UPS analysis is obtained (Figure S21d-f). The

PAA/TiO_2–NC reaches a voltage (about 345 mV) at a 1100 ml h^{-1} Ar/H_2O steam injection rate with light illumination, confirming the generation of hydrovoltaic effect in PAA/TiO_2–NC generator (Figure S21g). The H_2 evolution amount of PAA/TiO_2–NC generator is 1.2 mmol at the Ar/H_2O steam injection rate of 1100 ml h^{-1}, and O_2 gas is detected to be 0.5 mmol in the first cycle as shown in Figure S21h, higher than the performance of PAA/TiO_2–NC in bulk water (Figure S21i), suggesting the generality of hydrovoltaic effect enhanced photocatalysis.

Note S7: Calculations of Pt–H binding energy change. According to the simple harmonic motion, and assuming the *H (vibrational) and the Pt surface (fixed) are connected by a spring [31]:

$$k = \frac{f}{c} = \frac{\frac{1}{2\pi}\sqrt{\frac{K}{m}}}{c} \tag{23}$$

where k is the Raman shift number; f is the frequency; c is the velocity of light; K is the spring constant; and m is the mass of *H. Assuming the vibrational amplitude, A is constant [31]:

$$\mathrm{HBE} = \frac{1}{2}kA^2 \tag{24}$$

Combining equations (24) and (25):

$$\mathrm{HBE} \propto k^2 \tag{25}$$

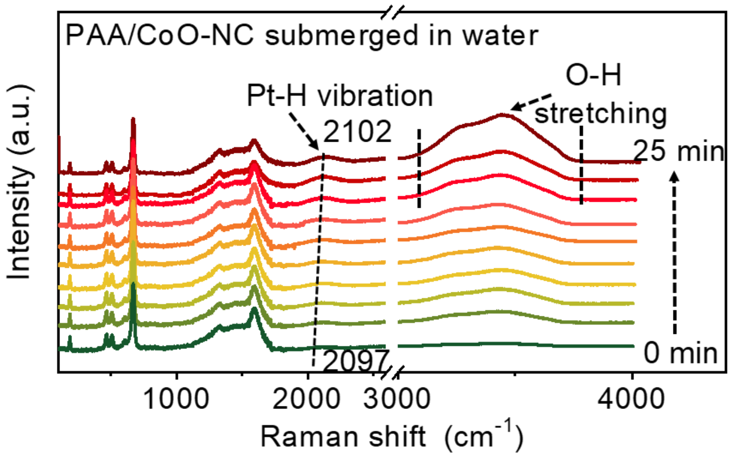

Figure S22. In situ Raman spectra of PAA/CoO–NC submerged in water without hydrovoltaic effect over time under light irradiation (AM 1.5G; 100 mW cm^2). Pt cocatalyst is loaded through a photodeposition method; A peak centred at 2097 cm^{-1} assigned to Pt–H vibration is detected in the situ recording Raman spectra of PAA/CoO–NC under light illumination and submerged in water. The Pt–H vibration gains less increase in intensity with time and has a smaller shift from 2097 cm^{-1} to 2102 cm^{-1} due to the vanished hydrovoltaic effect. The O–H stretching gains less increase in intensity over time, indicating that the interaction between water molecules and PAA/CoO–NC has reduced due to the bulky water and vanished hydrovoltaic effect.

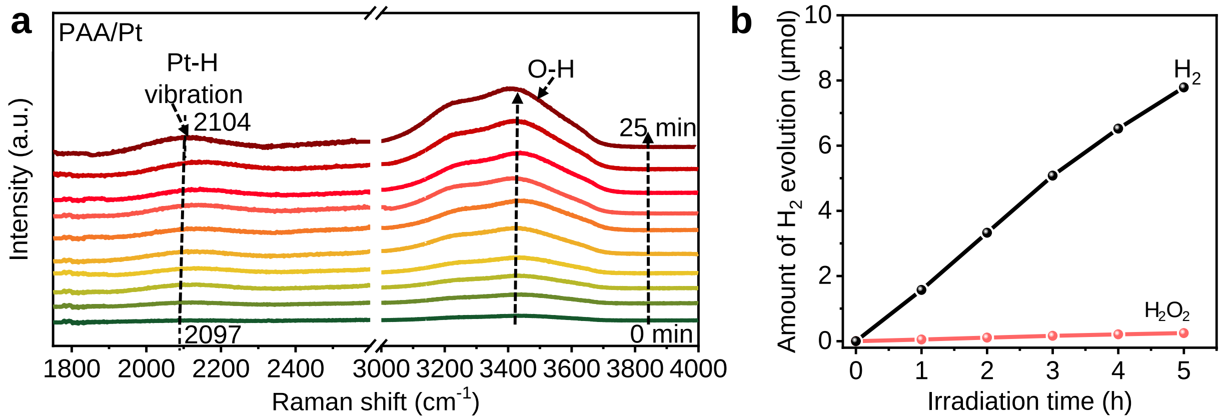

Figure S23. a In situ Raman spectra of the PAA/Pt surface over time with hydrovoltaic effect under light irradiation; **b** Time-dependent photocatalytic H_2 and H_2O_2 production of PAA/Pt; Pt cocatalyst is loaded using a photodeposition method; The light source is a solar simulator at AM 1.5G illumination (100 mW cm^{-2}).

We conducted photocatalytic performance measurement and measured the in situ Raman spectra on PAA/Pt at the 1100 ml h^{-1} Ar/H_2O steam injection under light illumination. As shown in Figure S23a, a peak centered at 2100 cm^{-1} assigned to Pt–H vibration for the in situ Raman spectra of PAA/Pt is observed, which gradually increases in intensity over time. The slight redshift of the Pt–H peak from 2097 to 2104 cm^{-1} and the slow increase in intensity O–H stretching at approximately 3200 and 3400 cm^{-1} over time, indicate a weak hydrovoltaic electric field in PAA/Pt. The H_2 evolution amount of PAA/Pt membrane is 7.8 μmol, and a trace amount of H_2O_2 of 0.3 μmol is detected due to the introduced hydrovoltaic effect of PAA and intrinsic hydrogen production characteristic of Pt (Figure S23b).

Note S8: Calculation of Schottky barrier height. The Schottky barrier height can be calculated by *I–V* curves according to thermionic emission theory, and the relationship between the applied voltage and the current can be expressed as [32]:

$$\mathrm{I} = I_0 \exp\left(\frac{qV}{\mathrm{nk}T}\right)\left[1 - \exp\left(\frac{-qV}{\mathrm{k}T}\right)\right] \tag{26}$$

where I_0, q, n, k, and T are the reverse saturation current, electronic charge, ideality factor, Boltzmann constant and temperature in Kelvin, respectively. For $V >$ 3kT/q, it can be simplified to [32]:

$$\mathrm{I} = I_0 \exp\left(\frac{qV}{\mathrm{nk}T}\right) \tag{27}$$

The reverse saturation current can be extracted by extrapolating the straight line of $\ln I$ to intercept the axis at zero voltage:

$$\ln I = \ln I_0 + \frac{qV}{\mathrm{nk}T} \tag{28}$$

and the reverse saturation is also theoretically determined by [32]:

$$I_0 = \mathrm{AA}^{*} T^2 \exp\left(\frac{-q\varphi_{\mathrm{b}}}{\mathrm{k}T}\right) \tag{29}$$

where A, A^{*}, and φ_{b} are the effective contact area, effect Richardson constant and zero-bias barrier height, respectively. Thus, the Schottky barrier height can be expressed as [32]:

$$\varphi_{\mathrm{b}} = \frac{\mathrm{k}T}{q} \ln\left(\frac{\mathrm{AA}^{*} T^2}{I_0}\right) \tag{30}$$

By plotting ln*I*–*V* curves, we can obtain the Schottky barrier height.

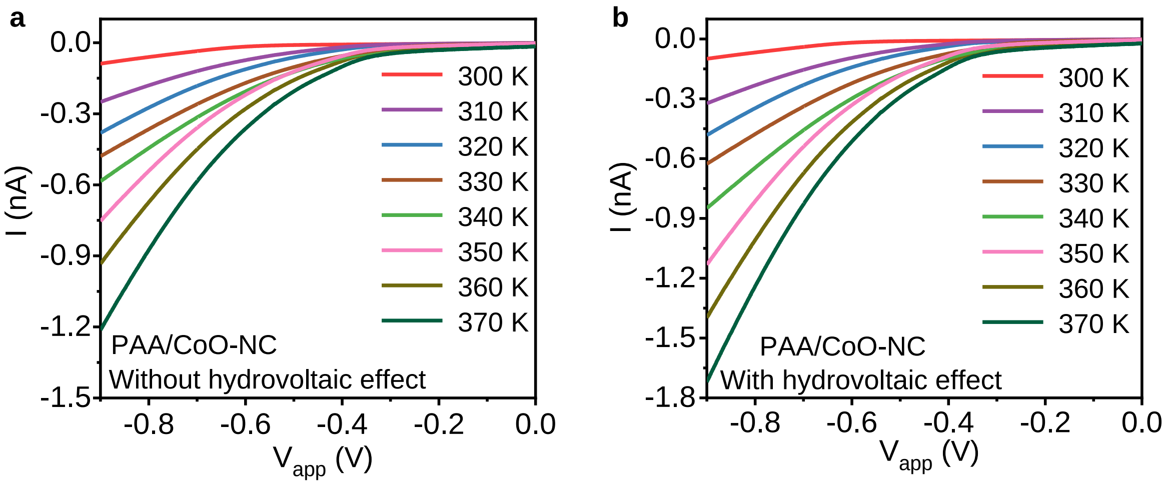

Figure S24. a *I–V* plots of PAA/CoO–NC in the temperature range of 300–370 K in the reverse bias region without hydrovoltaic effect; **b** *I–V* plots of PAA/CoO–NC in the temperature range of 300–370 K in the reverse bias region with hydrovoltaic effect. The properties of the potential barrier with hydrovoltaic effect were further evaluated based on a thermionic emission model and theory. The PAA/CoO–NC exhibited a higher current with hydrovoltaic effect.

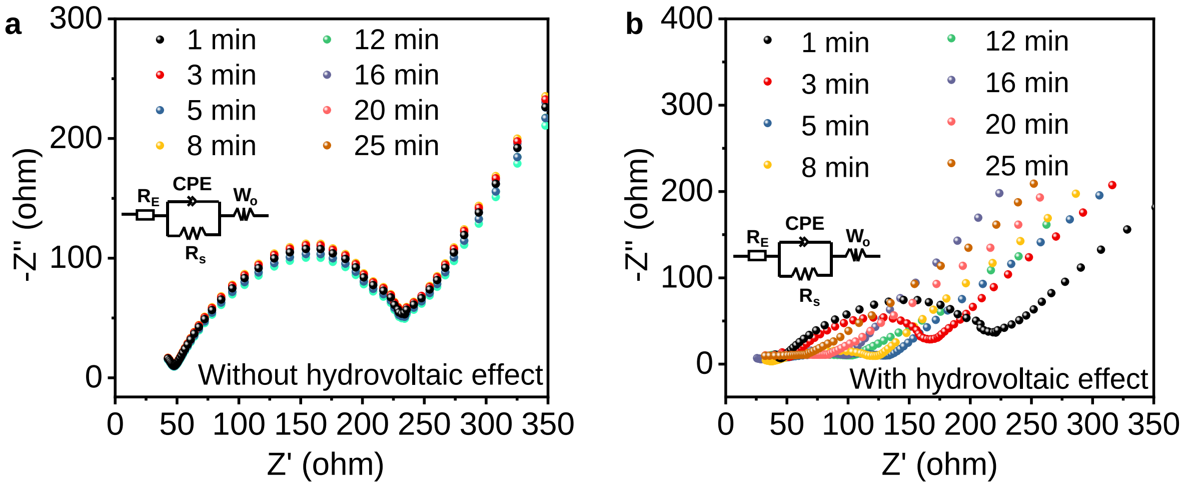

Figure S25. a EIS Nyquist plots of the electrochemical impedance spectra between the PAA/CoO–NC and moisture interaction as the time without hydrovoltaic effect; **b** EIS Nyquist plots of the electrochemical impedance spectra between the PAA/CoO–NC and moisture interaction as the time with hydrovoltaic effect. The inset shows the equivalent circuit, where R_E, R_S, CPE, and W_o represent the electrolyte resistance, electrode resistance, constant phase element and Warburg impedance, respectively [16].

EIS plots were performed on an AutoLab PGSTAT 204 electrochemical workstation. The frequency range was 0.1 Hz–10 kHz, and the magnitude of the modulation signal was set to be 0 V to avoid possible interface induced by the voltage. The equivalent circuit consists of an electrolyte resistance (R_E) in series connected with a combination of an electrode resistance (R_S) and a constant phase element (CPE) in parallel and serially connected with a Warburg impedance (W_o) [26]. EIS measurements were first applied to scrutinize the kinetic process of PAA/CoO–NC with or without hydrovoltaic effect. From these Nyquist plots, we find that the diameters of the semicircle gradually decrease as the interaction time of PAA/CoO–NC and moisture expands, indicating that the conductivity is improved with increasing hydrovoltaic effect. The EIS measurements of PAA/CoO–NC without hydrovoltaic effect show similar diameters of the semicircle with interaction time of PAA/CoO–NC and moisture longer, validating the nearly constant conductivity of PAA/CoO–NC without hydrovoltaic effect.

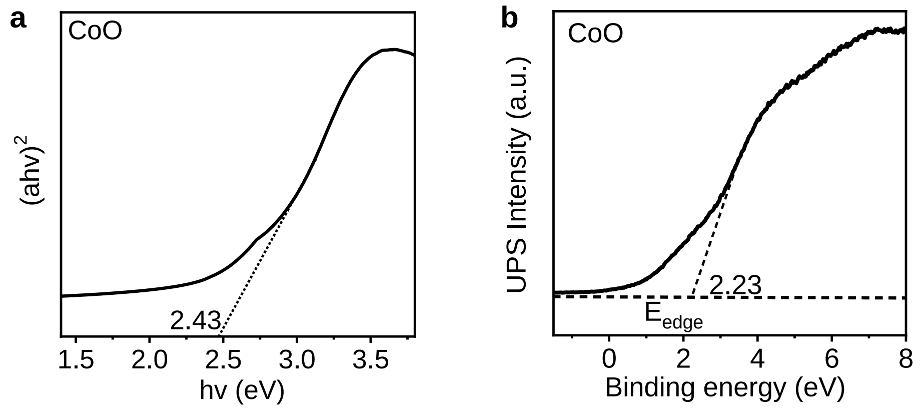

Figure S26. a Kubelka-Munk function vs. the energy of incident light plots; **b** UPS spectrum of the valence band energy region (E_{edge}) of CoO.

The secondary cut-off binding energy (E_{cutoff}) of CoO and NC can be determined as 16.83 and 16.79, respectively, by extrapolating the linear part to the baseline of the UPS spectra in Figure 5c. Based on the formula of work function (φ), the φ of CoO and NC can be calculated as 4.39 and 4.43 eV (vs. vacuum), respectively (hv of 21.22 eV: the excitation energy of the He I Source Gun). According to the linear intersection method [33-36], the E_{VBM} of CoO was calculated to be –6.62 eV (vs. vacuum) from hv+E_{Fermi}-E_{cutoff}. On the basis of the relationship between E_{vac} and the normal electrode potential (E^{θ}), E_{vac}=-E^{θ}-4.44 eV, the E_{VBM} of CoO was calculated to be 2.18 eV [37-41].

$$hv = E_{cutoff} + \varphi \tag{31}$$

$$E_{VBM} = E_{edge} + \varphi \tag{32}$$

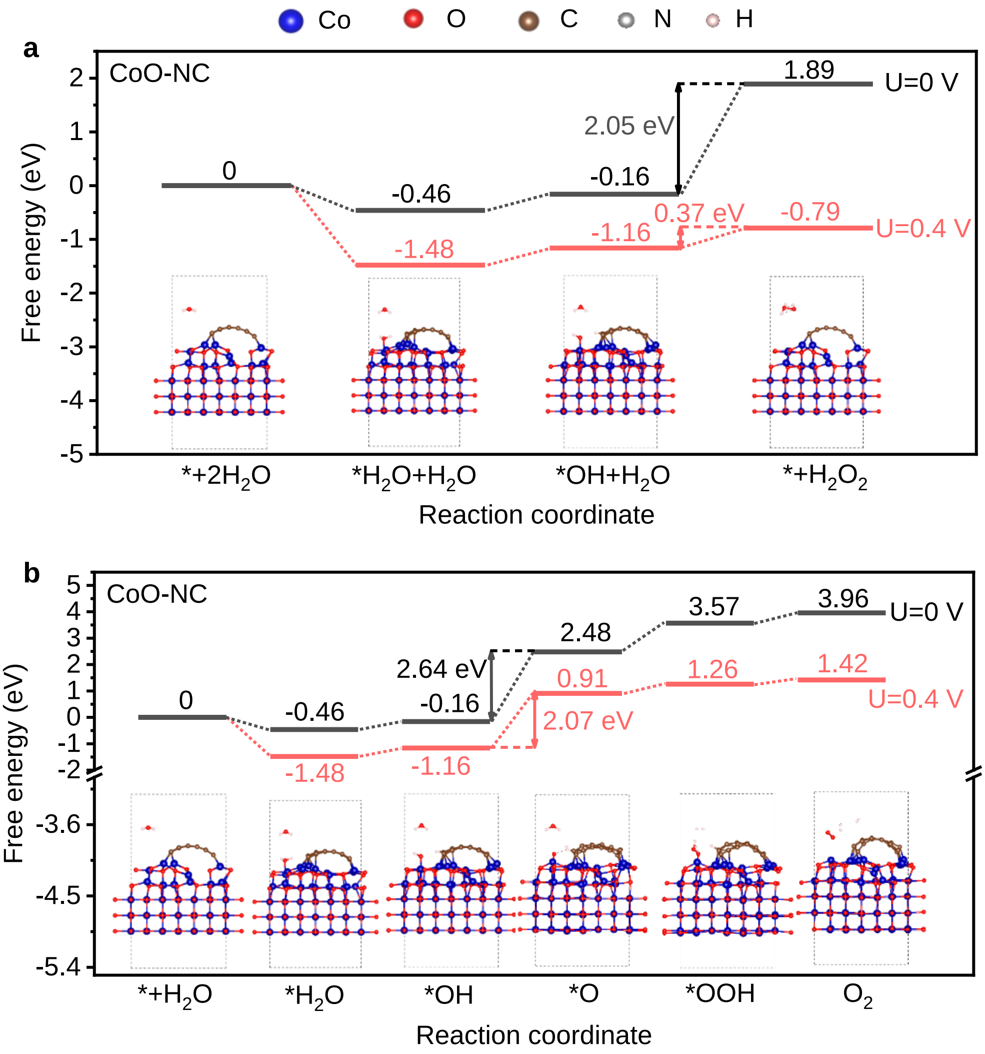

Figure S27. a DFT calculation of free energy diagram for the three-step H_2O_2 production process on CoO–NC with (U=0.4 V) or without (U=0 V) hydrovoltaic electric field and the corresponding adsorption geometries structures on CoO–NC; **b** DFT calculation of free energy diagram for O_2 production process on CoO–NC with (U=0.4 V) or without (U=0 V) hydrovoltaic electric field and the corresponding adsorption geometries structures on CoO–NC.

The adsorbed *OH stabilization on photocatalyst of the third step from *OH to *H_2O_2 is the most difficult thermodynamically in the entire H_2O_2 evolution reaction process (detail see Supporting Information of DFT Calculations). As shown in Figure S27a, the photocatalyst CoO–NC shows a much lower free energy barrier of 0.37 eV for H_2O_2 evolution process in the hydrovoltaic electric field than the intrinsic barrier of 2.05 eV on photocatalyst for the H_2O_2 production process, suggesting that the H_2O_2 reaction is much easier to occur on the system induced by the hydrovoltaic effect (The hydrovoltaic effect on energy barrier was investigated by setting a 0.4 V voltage on catalyst). In order

to further determine the photocatalyst for the H_2O_2 generation of high selectivity, rather than oxygen generation. We calculated the free-energy profiles for the O_2 evolution process, as shown in Figure S27b. The photocatalyst CoO–NC shows free energy barriers of 2.07 and 2.64 eV for the *O formation in O_2 production with or without hydrovoltaic effect, much higher than the limiting step barrier for the H_2O_2 generation. The corresponding water activation and adsorption models simulated on the photocatalyst CoO–NC were embeded below. As a consequence, the DFT-based calculations and energy band structure analysis indicate that the CoO–NC photocatalyst prefers to generate H_2O_2 rather than O_2 evolution.

Table S1. Co 2p XPS fitting data of CoO-NC and PAA/CoO-NC

Catalysts	Co $2p_{3/2}$			Co $2p_{1/2}$		
	Peak (eV)	FWHM	Area	Peak (eV)	FWHM	Area
CoO-NC	778.6	1.01	23098	794.1	1.08	10765
PAA/CoO-NC	777.6	1.06	19852	793.7	1.02	7894

Table S2. N 1s XPS fitting data of CoO-NC and PAA/CoO-NC

Catalysts	Pyridinic N			Co-N			Pyrrolic N		
	Peak	FWHM	Area	Peak	FWHM	Area	Peak	FWHM	Area
CoO-NC	398.7	1.03	35687	399.4	1.02	34672	401.2	1.09	12456
PAA/CoO-NC	398.6	1.07	37789	399.4	1.01	18558	401.1	1.03	11098

Table S3. O 1s XPS fitting data of CoO-NC and PAA/CoO-NC

Catalysts	Lattice oxygen			Hydroxyl		
	Peak (eV)	FWHM	Area	Peak (eV)	FWHM	Area
CoO-NC	530.3	1.03	22048	532.1	1.05	6765
PAA/CoO-NC	530.3	1.02	19842	531.9	1.02	16894

Table S4. Dependence of different wavelengths for H_2 generation in PAA/CoO-NC system and AQY calculation

Wavelengths (λ, nm)	H_2 evolved (mmol h^{-1})	Light power (mW)	AQY (%)
400	2.12	6.27	56.2
420	2.06	6.01	54.2
425	2.02	5.95	53.1
450	1.93	5.50	51.8
500	1.84	4.97	49.2
550	1.51	3.73	48.9
650	0.63	1.39	46.3

AQY calculation: Take AQY@400 nm of PAA/CoO-NC as an example:

$$N = \frac{\mathrm{S} \times P \times \mathrm{t} \times \lambda}{\mathrm{h} \times \mathrm{c}} = \frac{6.27 \times 10^{-3} \times 400 \times 10^{-9} \times 3600}{6.626 \times 10^{-34} \times 3 \times 10^{8}} = 0.454 \times 10^{20}$$

$$\mathrm{AQY} = \frac{2 \times n_{H_2} \times \mathrm{N_A}}{N} \times 100\%$$

$$= \frac{6.02 \times 10^{23} \times 2.12 \times 10^{-3} \times 2}{0.454 \times 10^{20}} \times 100\% = 56.2\%$$

Table S5. Summary of emerging methods in various electric power generation

Energy conversion unit	Open-circuit voltage	Short-circuit current or density	Power generation method	Ref.
Graphene oxide film after electric annealing	20 mV	5 μA cm^{-2}	Moisture induced electricity	10
Polypyrrole framework with anions gradient	60 mV	10 μA cm^{-2}	Moisture induced electricity	11
Nanostructured carbon materials	1 V	100 nA	Water-evaporation-induced electricity	12
Monolayer graphene	0.11 V	11 μA	Waving potential	13
Positively and negatively charged nanofluidic membrane	152.8 mV	/	Osmotic power	14
Graphene hydrogel membrane	/	2.23 nA	Streaming current	15
Pristine graphene oxide film	0.4 V	2–25 μA cm^{-2}	Moisture induced electricity	16
Al_2O_3 ceramic sheet	0.7 V	0.3 μA	Water-evaporation-induced electricity Based on liquid-solid triboelectric nanogenerators	17
Silicon nanowire arrays	400 mV	40 nA cm^{-2}	Water-evaporation-induced electricity	18
Asymmetric ionic nanofibrous aerogel	115 mV	15 nA cm^{-2}	Moisture induced electricity	19
Carbon nanoparticle	60 mV	3 nA cm^{-2}	Proton transport	20
GO framework	279 mV	3040 μA cm^{-2}	Proton transport	21
Polyoxometalates modified GO	42 mV	6.0 μA cm^{-2}	Proton transport	22
PAA/CoO–NC	402 mV	16.9 μA cm^{-2}	Moisture induced electricity	This work

Table S6. Comparison of photocatalytic performance for Co–based photocatalysts

Catalysts	Reaction conditions	H_2 evolution rate (μmol h^{-1})	H_2O_2 or O_2 production rate (μmol h^{-1})	AQY (%) for H_2 production	Ref.
PAA/CoO–NC	300 W Xe lamp, 0.05 g catalyst.	2420 (λ > 420 nm)	1105 H_2O_2 (λ > 420 nm)	56.2 @400 nm	This work
CoO–rGO	300 W Xe lamp, 0.02 g catalyst.	17.57 (λ > 420 nm)	11.3 O_2 (λ > 420 nm)	11.3 @420 nm	28
CoO–TS	300 W Xe lamp, 0.04 g catalyst	130.2 (λ > 420 nm)	55.6 H_2O_2 (λ > 420 nm)	8.3 @420 nm	29
CdS–Co–CoO_x/C	300 W Xe lamp, 0.1 g catalyst,	1997 (λ > 420 nm)	/	43.7 @420 nm	30
H–Ni–Co LDH	300 W Xe lamp, 0.1 g catalyst.	3.63 (λ > 420 nm)	1.80 O_2 (λ > 420 nm)	23 @380 nm	5
CdS/Ti_3C_2/CoO	300 W Xe lamp, 0.1 g catalyst,	134.46 (λ > 420 nm)	/	0.9 @380 nm	33
Co–CN	300 W Xe lamp, 0.001 g catalyst	12.63 (λ > 420 nm)	/	8.9 @380 nm	34
CoO–C wood	300 W Xe lamp, 0.05 g catalyst,	220.74 (λ > 420 nm)	/	50.1 @420 nm	35
CoP/Co_3O_4	300 W Xe lamp, 0.2 g catalyst,	344.9 (λ > 420 nm)	/	40.5 @420 nm	36
Co_1–phosphide/PCN	300 W Xe lamp, 0.05 g catalyst.	0.41 (λ > 300 nm)	0.20 H_2O_2 (λ > 300 nm)	4.6 @420 nm	37
$Cd_{0.5}Zn_{0.5}S$/CoO hybrid	300 W Xe lamp, 0.05 g catalyst,	89 (λ > 420 nm)	/	37.1 @420 nm	38
Co–MCM	300 W Xe lamp, 0.05 g catalyst	140 (λ > 420 nm)	/	12.3 @420 nm	39
Co–P	300 W Xe lamp, 0.05 g catalyst	15 (λ > 420 nm)	/	42.5 @430 nm	40
CoO_x/MoS_2	300 W Xe lamp, 0.05 g catalyst	370 (λ > 420 nm)	/	7.6 @420 nm	41

References

1. Wu, Q. et al. MOF-derived Co/CoO particles prepared by low temperature reduction for microwave absorption. *Chem. Eng. J.* **410**, 128378-128391 (2021).

2. Zhang, H. et al. Single atomic iron catalysts for oxygen reduction in acidic media: particle size control and thermal activation. *J. Am. Chem. Soc.* **139**, 14143-14149 (2017).

3. Sun, X. et al. High-performance single atom bifunctional oxygen catalysts derived from ZIF-67. *Nano Energy* **61**, 245-250 (2019).

4. Zhong, M. et al. Dually cross-linked single network poly(acrylic acid) hydrogels with superior mechanical properties and water absorbency. *Soft Matter* **12**, 5420-5428 (2016).

5. Wang, M. et al. A hydrogen-deficient nickel-cobalt double hydroxide for photocatalytic overall water splitting. *Angew. Chem. Int. Ed.* **132**, 11510-11515 (2020).

6. Ekspong, J. et al. Hydrogen evolution reaction activity of heterogeneous materials: a theoretical model. *J. Phys. Chem. C* **124**, 20911-20921 (2020).

7. Ma, Q. et al. Rational design of MOF-based hybrid nanomaterials for directly harvesting electric energy from water evaporation. *Adv. Mater.* **32**, 2003720-2003726 (2020).

8. Wang, X. et al. Hydrovoltaic technology: from mechanism to applications. *Chem. Soc. Rev.* **51**, 4902-4927 (2022).

9. Xiang, S. et al. A unique Co@CoO catalyst for hydrogenolysis of biomass derived 5-hydroxymethylfurfural to 2,5-dimethylfuran. *Nat. Commun.* **13**, 3657-3666 (2022).

10. Chen, L. G. et al. Ion seieving in graphene oxide membranes via cationic control of interlayer spacing. *Nature* **550**, 380-383 (2017).

11. Koltonow, A. R. et al. Two- dimensional nanofluidics. *Science* **351**, 1395-1396 (2016).

12. Zhao, F. et al. Direct power generation from a graphene oxide film under moisture. *Adv. Mater.* **27**, 4351-4357 (2015).

13. Xue, G. et al. Water-evaporation-induced electricity with nanostructured carbon materials. *Nat. Nanotechnol.* **12**, 317-321 (2017).

14. Yin, J. et al. Waving potential in graphene. *Nat. Commun.* **5**, 3582-3588 (2014).

15. Ji, J. et al. Osmotic power generation with positively and negatively charged 2D nanofluidic membrane pairs. *Adv. Funct. Mater.* **27**, 1603623-1603631 (2017).

16. Xu, T. et al. Electric power generation through the direct interaction of pristine graphene-oxide with water molecules. *Small* **14**, 1704473-1704480 (2018).

17. Cui, J. et al. Harvesting water-evaporation-induced electricity based on liquid–solid triboelectric nanogenerator. *Adv. Sci.* **9**, 2201586-2201594 (2022).

18. Qin, Y. et al. Constant electricity generation in nanostructured silicon by evaporation-driven water flow. *Angew. Chem. Int. Ed.* **59**, 10619-10625 (2020).

19. Yang, W. et al. Asymmetric ionic aerogel of biologic nanofibrils for harvesting electricity from moisture. *Nano Energy* **71**, 104610-104617 (2020).

20. Liu, K. et al. Induced potential in porous carbon films through water vapour absorption. *Angew. Chem. Int. Ed.* **55**, 8003-8010 (2016).

21. Zhao, F. et al. Highly efficient moisture-enabled electricity generation from graphene oxide frameworks. *Energy Environ. Sci.* **9**, 912-916 (2016).

22. Liu, J. et al. Moisture-enabled electricity generation from gradient polyoxometalates-modified sponge-like graphene oxide monolith. *J. Mater. Sci.* **54**, 4831-4841 (2019).

23. Huang, Y. et al. All-region-applicable, continuous power supply of graphene oxide composite.

Energy Environ. Sci. **12**, 1848-1856 (2019).

24. Liu, K. et al. Induced potential in porous carbon films through water vapor absorption. *Angew. Chem. Int. Ed.* **55**, 8003-8007 (2016).

25. Shen, D. et al. Self-powered wearable electronics based on moisture enabled electricity generation. *Adv. Mater.* **30**, 1705925-1705933 (2018).

26. Wang, M. et al. Red phosphorus/carbon nitride van der waals heterostructure for photocatalytic pure water splitting under wide spectrum light irradiation. *ACS Sustainable Chem. Eng.* **8**, 13459-13466 (2020).

27. Li, R. et al. Achieving overall water splitting using titanium dioxide-based photocatalysts of different phases. *Energy Environ. Sci.* **8**, 2377-2382 (2015).

28. Lin, Z. et al. Two-dimensional amorphous CoO photocatalyst for efficient overall water splitting with high stability. *J. Catal.* **372**, 299-310 (2019).

29. Zhu, M. et al. Efficient photocatalyst water splitting through titanium silicalite stabilized CoO nanodots. Nanoscale **11**, 15984-15990 (2019).

30. Ren, X. et al. Rational construction of dual cobalt active species encapsulated by ultrathin carbon matrix from MOF for boosting photocatalytic H_2 generation. *Appl. Catal. B Environ.* **286**, 119924-119934 (2021).

31. Wright, D. et al. Vibrational stark effects: ionic influence on local fields. *J. Phys. Chem. Lett.* **13**, 4905-4911 (2022).

32. Suyatin, D.B. et al. Strong schottky barrier reduction at Au-catalyst/GaAs nanowire interfaces by electric dipole formation and fermi-level unpinning. *Nat. Commun.* **5**, 3221-3229 (2014).

33. Ai, Z. et al. Construction of CdS@Ti_3C_2@CoO hierachical tandem p-n heterojunction for boosting

photocatalytic hydrogen production in pure water. *Chem. Eng. J.* **383**, 123130-123139 (2020).

34. Guo, P. et al. The synergistic effect of Co/CoO hybrid structure combined with biomass materials promotes photocatalytic hydrogen evolution. *Chem. Eng. J.* **420**, 130372-130383 (2021).

35. Guo, S. et al. Boosting photocatalytic hydrogen production from water by photothermally induced biphase systems. *Nat. Commun.* **12**, 1343-1353 (2021).

36. Peng, S. et al. CoP decorated with Co_3O_4 as a cocatalyst for enhanced photocatalytic hydrogen evolution via dye sensitization. *Appl. Surf. Sci.* **487**, 315-321 (2019).

37. Liu, W. et al. Single-site active cobalt-based photocatalyst with a long carrier lifetime for spontaneous overall water splitting. *Angew. Chem. Int. Ed.* **56**, 9312-9317 (2017).

38. Zhao, H. et al. A homojunction-heterojunction-homojunction scaffold boosts photocatalytic H_2 evolution over $Cd_{0.5}Zn_{0.5}S$/CoO hybrids. *J. Mater. Chem. A* **8**, 1955-1965 (2020).

39. Liu, X. et al. Confining Mo-activated CoS_x active sites within MCM-41 for highly efficient dye-sensitized photocatalytic H_2 evolution. *J. Colloid Interf. Sci.* **563**, 112-121 (2020).

40. Tian, B. et al. Supported black phosphorous nanosheets as hydrogen-evolving photocatalyst achieving 5.4% energy conversion efficiency at 353 K. *Nat. Commun.* **9**, 1397-1408 (2018).

41 Di, T. et al. Photodeposition of CoO_x and MoS_2 on CdS as dual cocatalysts for photocatalytic H_2 production. *J. Mater. Sci. Technol.* **124**, 209-216 (2022).
